# Electrochemical Wearable Biosensors and Bioelectronic Devices Based on Hydrogels: Mechanical Properties and Electrochemical Behavior

**DOI:** 10.3390/bios13080823

**Published:** 2023-08-15

**Authors:** Mohsen Saeidi, Hossein Chenani, Mina Orouji, MahsaSadat Adel Rastkhiz, Nafiseh Bolghanabadi, Shaghayegh Vakili, Zahra Mohamadnia, Amir Hatamie, Abdolreza (Arash) Simchi

**Affiliations:** 1 Department of Materials Science and Engineering, Sharif University of Technology, Tehran 14588-89694, Iran; hossein.chenani@sharif.edu (H.C.); mina.orouji@sharif.edu (M.O.); mahsa.adel78@sharif.edu (M.A.R.); n.bolghanabadi@sharif.edu (N.B.); 2 Polymer Research Laboratory, Department of Chemistry, Faculty of Science, University of Zanjan, Zanjan 45371-38791, Iran; shvakili71@gmail.com; 3 Department of Chemistry, Institute for Advanced Studies in Basic Science (IASBS), Gava Zang, Zanjan 45137-66731, Iran; z.mohamadnia@iasbs.ac.ir; 4 Department of Chemistry and Molecular Biology, University of Gothenburg, 405 30 Gothenburg, Sweden; 5 Institute for Nanoscience and Nanotechnology, Sharif University of Technology, Tehran 14588-89694, Iran

**Keywords:** flexible biosensors, electroactive hydrogel, biocompatible polymer, electrochemistry, mechanical behavior

## Abstract

Hydrogel-based wearable electrochemical biosensors (HWEBs) are emerging biomedical devices that have recently received immense interest. The exceptional properties of HWEBs include excellent biocompatibility with hydrophilic nature, high porosity, tailorable permeability, the capability of reliable and accurate detection of disease biomarkers, suitable device–human interface, facile adjustability, and stimuli responsive to the nanofiller materials. Although the biomimetic three-dimensional hydrogels can immobilize bioreceptors, such as enzymes and aptamers, without any loss in their activities. However, most HWEBs suffer from low mechanical strength and electrical conductivity. Many studies have been performed on emerging electroactive nanofillers, including biomacromolecules, carbon-based materials, and inorganic and organic nanomaterials, to tackle these issues. Non-conductive hydrogels and even conductive hydrogels may be modified by nanofillers, as well as redox species. All these modifications have led to the design and development of efficient nanocomposites as electrochemical biosensors. In this review, both conductive-based and non-conductive-based hydrogels derived from natural and synthetic polymers are systematically reviewed. The main synthesis methods and characterization techniques are addressed. The mechanical properties and electrochemical behavior of HWEBs are discussed in detail. Finally, the prospects and potential applications of HWEBs in biosensing, healthcare monitoring, and clinical diagnostics are highlighted.

## 1. Introduction

The growing popularity of wearable biosensors in healthcare management stems from their capacity to continuously and instantly gather physiological data by means of noninvasive analysis of biochemical markers present in biofluids, such as sweat, tears, saliva, and interstitial fluid [1]. Flexible and stretchable biosensors are also gaining attention due to their enhanced signal validity, patient comfort, and excellent mechanical properties, which allow for effective skin–device interface coupling and skin monitoring [2].

Wearable biosensors made of various materials are being developed for non-invasive, wireless, and consistent human health monitoring, which can help diagnose diseases in their preliminary stage, potentially reducing the economic burden caused by chronic and acute diseases on humans [3,4]. Some of these applications include cardiovascular disease monitoring [5], biological signals monitoring, such as glucose [6], lactate [7], pH [8], and body electrolytes [9], as well as recording various physiological parameters, including heart rate [10], electrocardiogram signals [11], body temperature [12], and blood oxygen levels [13] in real-time.

Biosensors consist of different components and sensing mechanisms that define biointerfaces. There are various types of wearable biosensors available, including chemical and physical biosensors, which are based on their sensing platforms. Chemical biosensors use chemical reactions to detect and quantify analytes in biological samples, while physical biosensors employ mechanical and optical properties for the same purpose. Some of the most common sensing mechanisms used in wearable biosensors are electrochemical, mechanical, and optical biosensing [14]. Wearable electrochemical biosensors (WEBs), in particular, have demonstrated promising results in clinical applications, particularly for continuous monitoring of biological signals [15]. These biosensors have been designed to have a fast response time and a high sensitivity, making them ideal for detecting low levels of analytes in a sample. Additionally, they are portable and can be used in a variety of settings, from laboratories to remote locations.

Recently, hydrogel-based wearable electrochemical biosensors (HWEBs) as an innovative technology are becoming increasingly popular since they take advantage of hydrogels and WEB devices [16]. HWEBs are advanced sensing devices using hydrogel materials as the platforms for immobilizing biorecognition elements [17,18]. The high selectivity and sensitivity of HWEBs make them a promising alternative to traditional analytical methods [19].

Hydrogels as soft, biocompatible, biodegradable and usually hydrophilic materials with a weak mechanical strength but acceptable elasticity resembling human tissues can be simply integrated into wearable devices to offer a non-invasive and flexible platform for continuous monitoring [20]. Hydrogels with a unique structure, including a three-dimensional (3D) network of crosslinked polymers, can absorb and retain large amounts of water in their interstitial spaces whilst maintaining their structural integrity in the swollen state [21]. The hydrogel surface can also be functionalized by various functional groups to enhance their specificity toward the target analyte [22]. In addition, hydrogels can be functionalized with various biorecognition elements, including enzymes, antibodies, and nucleic acids, to specifically detect the analyte of interest [23]. Most hydrogels do not demonstrate high electrical conductivity by their inherent nature. However, their conductivity can be improved through certain methods, such as hybridization with conductive materials and functionalization with redox and biomolecule species, which are elaborated in Section 2.3.1 and Section 2.3.2.

HWEBs offer a unique combination of mechanical and chemical stability, biocompatibility, and high swelling capacity, which is essential for detecting biological analytes in complex environments [22,24]. The hydrogel surface can also be functionalized by various functional groups to enhance their specificity toward the target analyte [22]. The immobilized biomolecules in HWEBs can catalyze a corresponding redox reaction, leading to a change in the current, potential, or impedance at the electrode surface [22,25]. The softness of HWEBs can release mechanical stress on the biological elements, leading to more stable and reliable biosensors [26]. However, the development and implementation of these biosensors are limited by the availability of suitable platforms that can provide the necessary functionality and performance.

This review provides a comprehensive overview of the advancements, challenges, and opportunities in the field of hydrogel-based wearable electrochemical biosensors. Our analysis covers various perspectives, including materials, properties, platforms, and applications. Specifically, we highlight the electrochemical and mechanical properties of HWEBs, while also discussing other critical properties briefly. We examine the factors that impact the performance of HWEBs, such as hydrogel materials and incorporated electroactive materials. Furthermore, we showcase the broad range of applications for HWEBs in healthcare management. This review is a valuable resource for researchers, engineers, and clinicians seeking to deepen their understanding of HWEBs and identify areas for future development.

## 2. Materials for Electrochemical Wearable Biosensors

### 2.1. Hydrogels

Hydrogel consists of a 3D hydrophilic polymer network with a high percentage of water, and its porous structure has provided the basis for the penetration of free ions [27]. Due to these characteristics, hydrogels have wide applications in sensors, biomedicine, and biomaterials [28,29]. Hydrogels can be categorized from various viewpoints, such as their origin (natural or synthetic polymer), composition (homopolymer network, copolymer network, and cross-linking network), method of cross-linking (physical or chemical), charge (anionic or cationic), and degradability (biodegradable and biocompatible) [30].

Hydrogels are wonderful materials for many applications due to their ability to absorb water, soft structure, biocompatibility, and structural similarity to the extracellular matrix. However, conventional hydrogels have limited applications due to their lack of conductivity and water evaporation [31]. Researchers have explored different crosslinking methods and polymer structures to improve the water retention capacity of hydrogels. This involves designing hydrogels with interconnected networks that can better hold water molecules, thereby reducing evaporation. The addition of conductive polymers and fillers to the hydrogel matrix enables the creation of composite materials possessing distinct physical and mechanical characteristics, which can be employed in various specialized biological applications.

#### 2.1.1. Conductive Polymer Hydrogels

**Figure 1:**
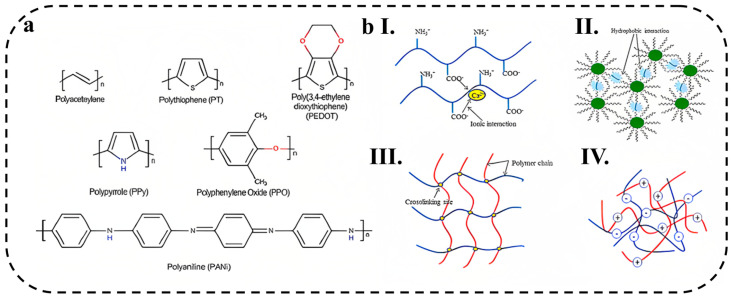
(**a**) Chemical structures of conductive polymers. Reprinted with permission from ACS from ref. [40], (the schematic representation of various physical cross-linking mechanisms observed in hydrogels including (**bI**) ionic interaction, (**bII**) hydrophobic interaction, (**bIII**) cross-linking junction formed through cooling, and (**bIV**) complex coacervation. Reprinted with permission from ACS from ref. [41].

Conductive hydrogels were first developed in 1994 by adding conductive components to conventional hydrogels [32]. Conductive hydrogels are one of the most effective materials that can be used to reproduce the electrical and biological properties of conductive body tissues. Most hydrogels are natural insulators, which can be converted into conductive hydrogels by adding various fillers, such as carbon metal particles and fibers (carbon nanotubes (CNT) and graphene) [33,34], metal nanoparticles [35], and conductive polymers (such as polythiophene, polyaniline (PANi), and polypyrrole (PPy)) [36,37]. Metal nanoparticles and carbon-based materials have been widely reported in biomedical applications due to their electrical properties. However, long-term cytotoxicity has limited their applications, and researchers have turned their attention to replacing them with polymeric materials [38]. Polymer materials are used in different forms, such as particles, core-shell, micelles, and dendrimers. Conductive polymer hydrogels are a good candidate for numerous applications, such as tissue engineering, drug delivery systems, biosensors, solar cells, implants, and biomedicine [39] due to the common characteristics of organic conductors and polymeric materials. Some examples of conductive polymers are shown in Figure 1a.

*PANi-Incorporated Hydrogels*

Polyaniline has garnered significant interest among conductive polymers due to its favorable characteristics, including its straightforward synthesis, affordability, a broad range of applications, and high efficiency of polymerization. The first preparation of polyaniline occurred in 1835 through the anodic oxidation of aniline on a platinum electrode, resulting in the formation of a dark brown precipitate in an aqueous solution of sulfuric acid. Similar findings were reported for the anodic oxidation of aniline in a hydrochloric acid aqueous solution [42].

The conductivity of PANi is due to the movement of electrons, which is controlled through the transfer of protons, and the presence of water plays an important role in its conductivity. Polyaniline has a unique electronic conduction mechanism among other conductive polymers; many of these unusual properties are due to the inherent A-B (head-to-tail) configuration of the polymer, whereas most other conducting polymers are A-A (head-to-head) [43].

Jung and co-workers [44] developed an electrochemical sensing electrode based on polyaniline/hemin/reduced graphite oxide for the simultaneous determination of ascorbic acid (AA). The electrode demonstrated the capability to detect AA within the concentration range of 100–700 μM and exhibited a sensitivity of 90 μA mM^−1^. Nonetheless, the primary hurdle in PANi-based electrochemical sensors designed for AA detection lies in facilitating electron transfer and achieving a substantial electroactive surface area to enhance their sensing capabilities. To solve this challenge, Zhang et al. [45] prepared a non-enzymatic electrochemical sensor based on PANi film doped with phytic acid. The sensor was developed for monitoring the levels of AA in sweat across a broad range of concentrations (0.5–500 μM). The sensor exhibited a high sensitivity (665.5 and 326.2 μA mM^−1^ cm^−2^) and a low detection limit (0.17 μM) compared to the ascorbic acid present in sweat. The addition of phytate enhanced the electrical conductivity of the sensor’s film by promoting electron transfer between PANi chains, thereby improving its electrochemical properties for the detection of ascorbic acid.

*PPy-Incorporated Hydrogels*

Pyrrole was first polymerized in 1916 by the oxidation of pyrrole with H_2_O_2_. Polypyrrole has received much attention because of its biocompatibility, ease of polymerization, and chemical stability. Like many intrinsically conductive polymers that are prepared electrochemically by anodic oxidation, PPy is made from the oxidation of pyrrole or substituted pyrrole monomers [46]. Due to the good intrinsic properties of polypyrrole, this conductive polymer has many applications in batteries, electrochemical sensors, conductive textiles and fabrics, and drug delivery systems [47]. However, due to the insolubility of pyrrole and polypyrrole in water, the preparation of conductive PPy-incorporated hydrogels is still a big challenge. In a study by Wang et al. [48], water-soluble PPy was synthesized and utilized for the fabrication of conductive hydrogels incorporating chitosan, acrylamide, and cucurbituril. The resulting hydrogel demonstrated favorable mechanical properties, with a fracture strain of 2149.17% and a mechanical strength of 215.48 kPa. Additionally, the hydrogel exhibited strong adhesion capabilities (~51.54 kPa), high conductivity (0.534 S m^−1^), and biocompatibility, making it suitable for applications involving bodily movements and the sensing of physiological signals.

*PEDOT-Incorporated Hydrogels*

Poly(3,4-ethylenedioxythiophene) (PEDOT) is one of the conductive polymers, which is the most widely used thiophene derivative and has poor water solubility, which limits its application. To solve this problem, a mixture of PEDOT and polystyrene sulfonate (PSS) is used. Compared to other conducting polymers, PEDOT has advantages, such as long-term stability, high conductivity, easy synthesis method, suitable compatibility with other materials, biocompatibility, and low toxicity [49]. Gao et al. [50] developed a microfluidic-based electrochemical sensor for wearable applications. The sensor was created by integrating a conductive PEDOT: PSS hydrogel with carbon screen-printed electrodes (CPE). To enhance stability, the working electrode was modified using the electro-polymerization technique, resulting in increased adhesion between the PEDOT:PSS hydrogel and the electrodes. The flexible electrochemical sensor exhibited excellent properties, such as favorable conductivity and a large electroactive surface area, attributed to the PEDOT:PSS hydrogel. The sensor was specifically designed for accurate and sensitive detection of uric acid (UA) in sweat. Notably, the fabricated sensor demonstrated an exceptionally high sensitivity of 0.875 µA µM^−1^ cm^−2^ and a low limit of detection (LOD) of less than 1.2 µM (S/N = 3). These impressive characteristics make the sensor a promising device for non-invasive monitoring of human physiology and personalized healthcare applications. In another research that was conducted on PEDOT:PSS conductive hydrogel, Zhang et al. [51] introduced an innovative electrochemical biosensor that utilizes a PEDOT:PSS conductive hydrogel incorporated with Prussian blue nanoparticles (PBNPs) for the detection of glucose in the body. The biosensor exhibited a LOD of 0.85 μM and a remarkable sensitivity of up to 340.1 μA mM^−1^, making it suitable for monitoring glucose levels in diabetic individuals.

*Other Materials*

Polystyrene, polyfluorene, polyphenylene, and polyphosphazene are other commonly used conductive polymers that are added to hydrogels to produce conductivity. Depending on the preparation methods, conducting polymers can be widely tuned in terms of electrical properties, mechanical properties, and performance and used for various applications [52].

#### 2.1.2. Ionic Conductive Polymer Hydrogels

The 3D porous structure of hydrogels is due to the major contribution of water. The presence of this 3D network filled with water leads to the migration of free ions (protons, hydroxyls, etc.) in the hydrogel space and ion conduction. Ionic conducting hydrogels are used for applications such as electrochemical sensors and ionic skin sensors due to their excellent biocompatibility, inherent flexibility, adhesiveness, self-healing, and interesting electrochemical properties [53]. Ionic conductive hydrogels exhibit remarkable adaptability and exceptional responsiveness, rendering them highly suitable for utilization as wearable electronic devices in healthcare diagnostics. Their exceptional flexibility and heightened sensitivity make them promising contenders for the real-time monitoring of human body movements. Xu et al. [54] designed a super-stretchable and recoverable double network polymer hydrogel (SA-Zn): ZnSO_4_/sodium alginate/poly(acrylic-acrylamide) ionic conductive hydrogel. SA-Zn hydrogel displayed good conductivity and high sensitivity due to its hydrophilic interaction and zwitterionic structure. When combined with a WiFi transmitter, this hydrogel can be used as a wireless wearable electronic sensor with high sensitivity, fast response, reusable recovery and good identification. This ion-conducting hydrogel showed great potential for human body motion detection.

#### 2.1.3. Crosslinking Mechanisms

*Covalent Crosslinking*

Covalent crosslinking involves the formation of a polymer network by forming covalent bonds between polymer chains, as shown in Figure 1b. In this regard, various chemical reactions, including irradiation of vinyl polymers, cross-linking of small molecules, polymer–polymer crosslinking, enzyme-mediated reaction, and click reactions, have been used to prepare hydrogels through the chemical crosslinking [55].

*Supramolecular Crosslinking*

Supramolecular chemistry is described by noncovalent bonding between molecules, including hydrogen bonding, metal coordination, host–guest complexation, π–π stacking, and electrostatic interactions. The hydrogel system based on supramolecular interaction can be used in a wide range of applications, including the design and synthesis of catalysts and sensors, due to the supramolecular interactions that make it more adaptable and flexible [56].

*Physical Crosslinking*

The hydrogels containing physical crosslinking have non-covalent bonds between the chains, and these interactions are responsible for binding. Intermolecular forces exist for physical cross-linking, which include hydrogen bonds, metal-ligand coordination, host-guest interaction, ionic interaction, stereo-complexation, and self-assembly [41], which are shown in Figure 1b. Ionic cross-linking is a physical crosslinking that usually occurs between two oppositely charged molecules or polyelectrolytes. In ionic interactions, hydrogels can be cross-linked under mild conditions, at room temperature and physiological pH. For example, Liu et al. [57] prepared high-performance polyvinyl alcohol (PVA)/glycerol/sodium alginate (SA)/CaCl_2_ (PGSC) ionic hydrogel sensors with dual physically cross-linked networks. The main cross-linked network between glycerol and PVA and the second network through ionic cross-linking between Ca^2+^ ions and the carboxyl group of sodium alginate (SA) improved the mechanical properties of the hydrogel (maximum strain 816%, maximum stress 2.29 MPa). The obtained hydrogel showed high conductivity (2.08 × 10^−2^ S cm^−1^) and high sensitivity (GF = 2.68 at 500% strain) to accurately monitor various activities of the human body. Ko et al. [58] developed a double-network hydrogel-based strain sensor comprising vinyl hybrid silica nanoparticles (VSNPs)/polyacrylamide(PAAm)/alginate. Physical cross-linking among PAAm chains and covalent cross-linking between PAAm, alginate, and N,N’-methylenebisacrylamide chains improved mechanical properties. The obtained hydrogel showed high sensitivity (GF = 1.73 up to 100% strain), a low limit of detection of 0.4% and a negligible electrical hysteresis of 2.43%. The constructed sensor based on double-network hydrogels was successfully used to measure subtle to large deformations caused by human body movement. Chen et al. [59] reported a self-healing supramolecular hydrogel by introducing multiple hydrogen-bonding groups, 2-ureido-4[1H]-pyrimidinone (UPy), as a cross-linker into a polyaniline/poly(4-styrenesulfonate) (PANI/PSS) network. The hydrogel was composed of a negatively charged PSS supramolecular network cross-linked by UPy groups, where an interpenetrating conducting PANI network was formed by in situ polymerization and electrostatically interacted with PSS chains. The formed hydrogel showed conductivity of 13 S m^−1^ and high sensitivity (GF = 3.4 at 300% strain) for the detection of various human motions.

### 2.2. Hydrogel Composites

Introducing nanomaterials in a hydrogel substrate is an excellent route to produce an efficient HWEB with high sensitivity in a flexible substrate, making them more portable and biocompatible. Incorporating nanoparticles or composite materials into the hydrogel matrix can enhance its mechanical and electrical properties, reducing the impact of water evaporation. Nanomaterials, including metallic and non-metallic compounds, are mostly used to enhance the mechanical and electrochemical properties of HWEBs [60].

#### 2.2.1. Non-Metallic Nanomaterials

In recent years, non-metallic nanomaterials, such as MXenes, have been considered to develop HWEBs [61]. Such nanomaterials provide more active sites for the conjugation of biomolecules, such as antibodies and antigens, which enhance their catalytic activity and specificity toward the target molecule [62]. Furthermore, the flexibility and low thickness of some nanomaterials allow them to be put on the human skin or biological tissues and minimize surface tensions [63]. The following describes the structure and properties of such nanocomposites, as well as their applications in HWEBs.

*MXenes*

Mxenes, due to their metal layers, are remarkable among non-metallic compounds and can be varied from semiconductor to conductor upon their synthesis method. Ti_3_C_2_T_x_ is known as one of the most widely used MXenes with a high electron transferring rate and excellent chemical stability [64]. Lei et al. [65] constructed a multifunctional system using novel MXene/Prussian blue (TiC_2_T_x_/PB) nanocomposite modified with *glucose* oxidase enzyme (GOx) as a wearable wristband biosensing patch for glucose detection in sweat. The high electroactive surface area of the MXene and the formation of a solid–liquid–air three-phase interface on the porous surface of the hydrogel made excellent access of oxygen into the enzyme active layer, resulting in the significant activity of the enzyme with the electrochemical sensitivities of 35.3 and 11.4 µA mM^−1^ cm^−2^ for glucose and lactate, respectively.

**Figure 2:**
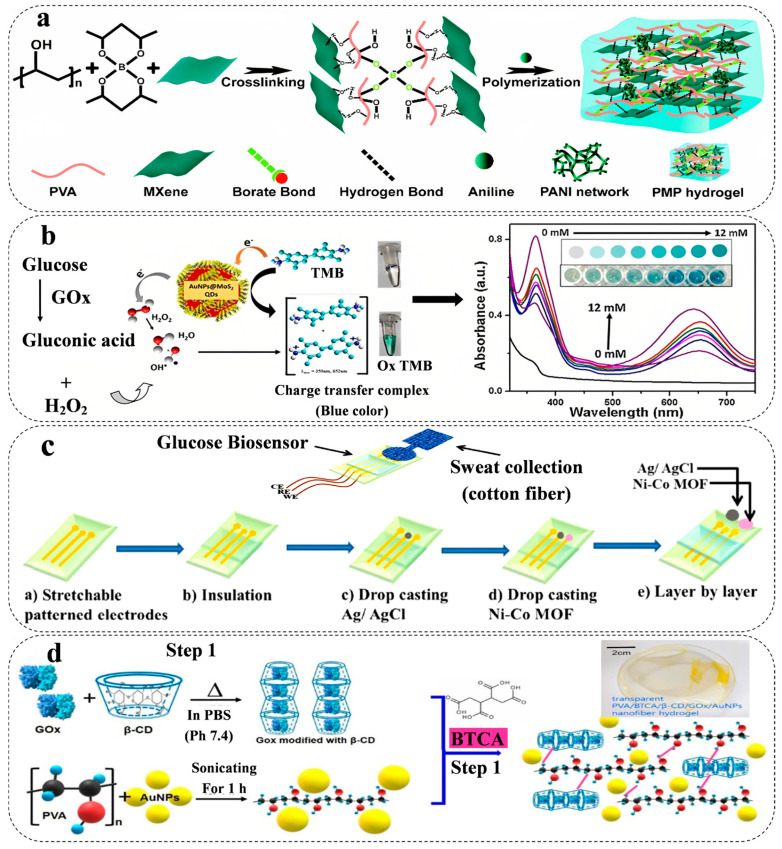
Schematic representation of (**a**) multifunctional polyvinyl alcohol/MXene/polyaniline hydrogel. Reprinted with permission from RSC from ref. [68], (**b**) AuNPs@MoS_2_-QDs composite with an agarose hydrogel-based stable visual platform in the presence of H_2_O_2_. The right diagram shows the UV–vis absorbance spectra of the hydrogel at various glucose levels (0, 2, 4, 5, 8, 10, 11, 12 mM) in serum. Reprinted with permission from Elsevier from ref. [69], (**c**) a flexible three-electrode system based on Ni-Co MOF-coated Au/PDMS hydrogel as a wearable electrochemical biosensor for glucose detection. Reprinted with permission from RSC from ref. [70], and (**d**) porous PVA/BTCA/β-CD/GOx/AuNPs composite integrated with PVA polymer nanofiber hydrogel synthesized by electrospinning method as a wearable glucose-responsive biosensor. Reprinted with permission from Nature from ref. [71].

However, the lack of stretchability and limited gelation ability of MXenes have negative effects on the mechanical performance of the hydrogel [66]. For wearable strain sensors, the chemical composition and interlayer space of MXenes should be wisely designed to reach desirable properties by adjusting the synthesis parameters and various functional groups on the surface of MXenes. The hydrogen bonding of MXenes with the matrix in the hydrogel creates compact layers [67,68]. Li et al. [68] reported an MXene/PVA nanocomposite for a wearable pressure sensor. The strong hydrogen bonding between PVA and MXene, as shown in (Figure 2a) with a tensile strength of 4.1 MPa, provides high mechanical properties along with high electrical conductivity (0.22 S m^−1^).

*Carbon-based Nanomaterials*

Carbon-based nanomaterials (CNMs) are one of the most widely applicable non-metallic nanomaterials in electrochemical systems. CNMs with special physical/chemical structure, various ranges of electrical conductivity, easy functionalization and adjustable specific surface area have been increasingly used in HWEBs [72].

Carbon quantum dots (CQDs) with massive active sites significantly reduce the interfacial impedance during sensing reactions and offer a high detection sensitivity [73]. Li et al. [74] examined how sulfurized GQDs affect the electrochemical activity of a PPy matrix. The thiol of the GQD protonated the nitrogen groups of PPy, making electrostatic interactions that resulted in the formation of a 3D hydrogel network. The findings demonstrated that GQDs acted as transport pathways, facilitating the faster diffusion of dissolved ions in the solution based on low frequencies, and possess a Warburg linear portion results (the conductivity increases from 7.41 × 10^−3^ to 0.5 S cm^−1^). Consequently, the GQDs enhanced the electrochemical and electrical activity of the hydrogel, which is enhanced by 49%. Currently, GQDs are widely used in electrochemical, optical, and sensor systems. Therefore, based on previous studies, the use of GQDs due to their non-toxicity and desirable electrochemical properties can be a suitable candidate in HWEBs.

CNTs with excellent characteristics such as high flexibility, excellent reactivity and good conductivity can be an ideal candidate for HWEBs [75]. Li et al. [76] introduced the use of nitrogen doped-CNTs in the hydrogel (concluding choline chloride as hydrogen bonding receptors and acrylamide and acrylic acid as hydrogen bonding donors) increased the electrical conductivity of the hydrogel by 4.2 times. The modification of carboxyl groups on CNTs improved the interactions of CNTs with the carboxyl groups in hydrogel and prevented CNTs from agglomerating on the hydrogel. The result indicated excellent compressive and strain strength, 4.29 MPa and 5.42 MPa, respectively, due to the interaction between N-CNTs and the matrix.

Graphene is a 2D CNM that consists of a single carbon layer with an SP^2^ hybrid hydrocarbon framework [77]. The structure of graphene poses limitations for its use in HWEBs, as its hydrophobic nature limits its interaction with biomolecules and biomaterials. However, graphene derivatives, such as graphene oxide (GO) and reduced graphene oxide (rGO), can address the issue [78]. Tang et al. [79] developed a redox sodium alginate-Pb^2+^-graphene oxide hydrogel as an ultrasensitive label-free electrochemical immunosensor detecting carbohydrate antigen 24-2. The presented electrode with the synergetic performance of Pb ions and GO exhibited a suitable electrocatalytic activity and stable electron transferring kinetics with a wide linear range from 0.005 to 500 U mL^−1^, the sensitivity of 32.98 μA (log_10_C_CA242_)^−1^ and low detection limit of 0.067 mU mL^−1^.

*Metal Oxide Nanoparticles*

Metal oxide nanomaterials, such as titanium dioxide (TiO_2_), Zinc oxide (ZnO), tin oxide (SnO_2_), and iron oxide (Fe_3_O_4_), have attracted significant attention as potential transducer materials due to their unique electrochemical and catalytic properties such as semiconductor properties, good specific surface area and stability. TiO_2_ nanoparticles in PANi hydrogel have also shown good electrocatalytic performance for glucose analysis by developing the stable p-n heterojunctions between the PANi (p-type) and the TiO_2_ nanoparticle (n-type) [80]. Wang et al. [81] introduced a one-pot strategy to synthesize a Fe_3_O_4_ nanoparticle-loaded 3D porous graphene (3D-GH) nanocomposite hydrogel by using GO sheets and hemin as a source of 3D graphene and Fe_3_O_4_, respectively. In this process, GO was concurrently self-assembled and reduced to form 3D-GH. Fe_3_O_4_ nanoparticle was formed on the surfaces of 3D-HG by chemical method. In fact, a synergetic effect between Fe_3_O_4_ nanoparticles with high catalytic activity and 3D-HG with high active site resulted in the outstanding peroxidase activity for colorimetric determination of glucose with a minimum detection limit of 0.8 μM, with the linear range of 5–500 μM.

*Transition Metal Dichalcogenides (TMDCs)*

TMDCs such as molybdenum disulfide (MoS_2_) and tungsten disulfide (WS_2_) show a wide range of conductivity (metallic/semi-metallic), a high specific surface area, making them ideal for use in HWEBs [82]. TMDCs can be used in electrochemical biosensors as both electrocatalysts and transducers [83]. As electrocatalysts, TMDCs can enhance the electrochemical activity of enzymes and other biomolecules that catalyze the oxidation or reduction of analytes in the solution [84]. For example, MoS_2_ has been shown to enhance the electrochemical activity of the enzyme (GOx), which catalyzes the oxidation of glucose to gluconic acid and hydrogen peroxide [85]. Vinita et al. [69] introduced a gold nanoparticle(AuNPs)@MoS_2_-QDs composite with agarose hydrogels demonstrating significant stability in different conditions (temperature and pH) to detect glucose in serum, saliva, and tear. The fascinating conductivity, biocompatibility, and, more importantly, marvelous catalytic properties of MoS_2_ (Figure 2b) exhibited a reaction rate of 10.6 μM s^−1^ with a detection limit of 0.068 μM.

*Metal-Organic Frameworks (MOFs)*

MOFs, porous materials composed of metal ions or clusters coordinated by organic ligands, have emerged as promising candidates for HWEBs due to their diversity in functional groups, high adsorption capacity, and catalytic properties [70]. For example, using the porous structure of Nickel (pyridine-2,6-dicarboxylic acid)-MOFs on the flexible carbon nanofiber hydrogel leading to increased electrocatalytic activity toward anodic glucose oxidation reaction with the maximum sensitivity of 9457.5 μA mM^−1^ cm^−2^ and minimum detection limit of 0.053 μM. As well as, results showed satisfying anti-inference activities to evaluate against various bio-compounds such as ascorbic acid, fructose, lactose, dopamine, sucrose, and uric acid [86]. Moreover, MOFs can be modified or combined with nanoparticles during the synthesis process because of the high specific surface and porosity. Shu et al. [70] introduced a Ni-Co MOF nanosheet-coated Au/PDMS film as a flexible glucose HWEB for sweat monitoring (Figure 2c). The findings demonstrated a robust electrochemical performance attributed to the porous structure of MOF and the enhanced conductivity of AuNPs, resulting in excellent electrocatalytic activity. The glucose detection capability of the sensor exhibited a substantial sensitivity of 205.1 μA mM^−1^ cm^−2^ and an expansive linear range spanning from 20 to 790 μM.

The use of MOFs in HWEBs is still in the early stages of development, but the potential applications are numerous. With further research and development, MOF-based biosensors could become an important tool for monitoring a wide range of biological analytes in various settings, from medical to environmental monitoring.

*Zeolites*

Zeolites are highly versatile materials that can be incorporated into biosensors for the selective detection of various biomarkers [87]. Zeolites are crystalline, microporous materials with high surface area and ion exchange capacity. The high surface area of zeolites provides a large number of binding sites for biomolecules, which can be used for the selective detection of various analytes in biological fluids such as blood, sweat, and tears [88]. When hydrogels are combined with zeolites, they can provide a stable and biocompatible platform for the immobilization of enzymes and other biomolecules [89]. The high-water content of hydrogels ensures that the biomolecules remain hydrated and active, while the zeolites provide porous support for the immobilization of enzymes and other biomolecules [90]. This combination of hydrogels and zeolites has led to the development of highly sensitive and selective electrochemical biosensors for a wide range of analytes. Panic et al. [91] developed the PMAA-MFI Zeolite composite hydrogel and investigated the effect of zeolite on the mechanical properties of the hydrogel. Using zeolite in hydrogel increased cross-linking density along with the strong modulus. Additionally, SEM results indicated the uniform distribution without the aggregation of zeolite crystalline, which emphasized improving mechanical performance and decreasing the values of the swelling degree.

#### 2.2.2. Metallic Nanomaterial

Metallic nanomaterials have emerged as promising candidates for use in biosensors due to their unique physicochemical properties [92]. The excellent conductivity, small size, and large surface area of metallic nanoparticles allow for enhanced sensitivity and selectivity in biosensing applications [93].

AuNPs have been shown to improve the stability and sensitivity of biosensors by providing a high surface area for immobilizing biological molecules, such as enzymes or antibodies, and facilitating electron transfer at the electrode surface [94]. A transparent nanofiber hydrogel patch capable of glucose-responsive behavior was successfully developed for continuous and non-invasive glucose monitoring from interstitial fluid (ISF). The nanofiber hydrogels, composed of PVA/BTCA/β-CD/GOx/AuNPs, were synthesized via electro-spinning, resulting in a porous structure with a notably high specific surface area (as depicted in Figure 2d). These hydrogels exhibited exceptional electrochemical and mechanical properties, including a tensile strength of 5.33 MPa. Notably, the hydrogels displayed high enzyme activity (76.3%), a considerable sensitivity of 47.2 μA mM^−1^, a low LOD of 0.01 mM, and a rapid response time of less than 15 s. Additionally, the hydrogels demonstrated flexibility, biocompatibility, and a high absorptivity [71].

In addition to AuNPs, silver nanoparticles (AgNPs) have also been explored, which exhibit unique physicochemical properties, such as high conductivity and antibacterial and cytotoxic efficacy, and can be harnessed to improve the performance of biosensors [95]. Zhang et al. [96] developed a AgNPs@gallic acid-modified collagen/poly(acrylic acid) hydrogel (AgNP@GCOL/PAA). Results indicated that the presence of AgNPs enhanced the electrical conductivity from 1.4 to 6.3 mS cm^−1^. Furthermore, the hydrogel without AgNPs did not show any antimicrobial ability, while the antibacterial ability of hydrogel increased by increasing the content of AgNPs, of which approximately 65% of S. aureus and 50% of E. coli were killed in the presence of AgNPs.

### 2.3. Hydrogel Functionalization

The performance of HWEBs can be limited by their sensitivity, selectivity, and stability [97]. To overcome these limitations, new strategies are being developed to improve the HWEB performance. The properties of hydrogels can be modified by incorporating functional groups into the polymer network [98] or by coating the hydrogel surface with specific molecules, such as redox species [99] or biomolecule species [100].

The first approach for hydrogel functionalization is the incorporation of nanoparticles into the hydrogel matrix, which causes enhancement in properties, for example, increasing mechanical strength and responsiveness to external stimuli [51,101]. The incorporation of conductive polymers into the hydrogel matrix is the second approach for hydrogel functionalization, which can enhance the electrical conductivity of the hydrogel and improve the sensing performance of the biosensor [102]. The third strategy for hydrogel functionalization is the introduction of chemical or biological moieties into the hydrogel structure, which can be achieved by covalent or non-covalent binding of functional groups or by incorporation of bioactive molecules [100]. Chemical modification of hydrogels is the next functionalization approach involving the incorporation of functional groups into the polymer network, which can be done by copolymerization with monomers containing functional groups [103]. By strategically introducing specific biological components into the HWEBs’ structure, it becomes possible to achieve and regulate their structures, compositions, biological functions, biodegradability, and mechanical stability. This spatial functionalization approach ensures that the hydrogels possess the desired characteristics and can effectively respond to targeted stimuli [104]. Amine (-NH_2_) group is a functional group that can be involved in the covalent bonding of biomolecules like DNA probes to hydrogel substrates [105]. This characteristic plays a vital role in the detection and sensing of specific target substances in wearable biosensors, leading to improved sensitivity and lower limits of detection [105]. Another widely utilized functional group in hydrogel-based biosensors is the carboxylic (-COOH) group [106]. The addition of carboxylic acid-containing monomers, like acrylic acid, allows for the introduction of functional groups into the hydrogels [106]. When the hydrogels have polymer chains that are grafted with carboxyl groups, they can effectively immobilize biomolecules through highly specific interactions and enhance the biosensor’s response, enabling accurate and reliable detection of target analytes [106,107]. Furthermore, the hydroxyl (-OH) group can play a significant role in the hydrogel functionalization [108]. Hydroxyl groups offer opportunities for additional chemical modifications or crosslinking reactions, which can improve the mechanical and electrochemical properties of the HWEBs [105,108]. The last method for hydrogel functionalization is physical adsorption, which entails the attachment of specific molecules onto the hydrogel surface through non-covalent interactions like hydrogen bonding, electrostatic interactions, and van der Waals forces. This technique is straightforward and offers the advantage of enabling the immobilization of diverse biomolecules onto the hydrogel surface [109,110].

#### 2.3.1. Redox Species

Hydrogel functionalization with redox species (such as Prussian blue nano/microstructures and metallic particles) is a promising strategy for developing HWEBs. It can provide special properties, including electrical conductivity and redox activity [51,111]. Functionalization of hydrogels with redox species can be achieved by incorporating them into the hydrogel matrix [51] or by grafting them onto the hydrogel [112]. One approach to incorporating redox species is to use redox-active monomers [113]. Redox species can also be immobilized on the electrode surface, allowing for direct electron transfer between the analyte and the electrode. This technique eliminates the need for a mediator [114].

**Figure 3:**
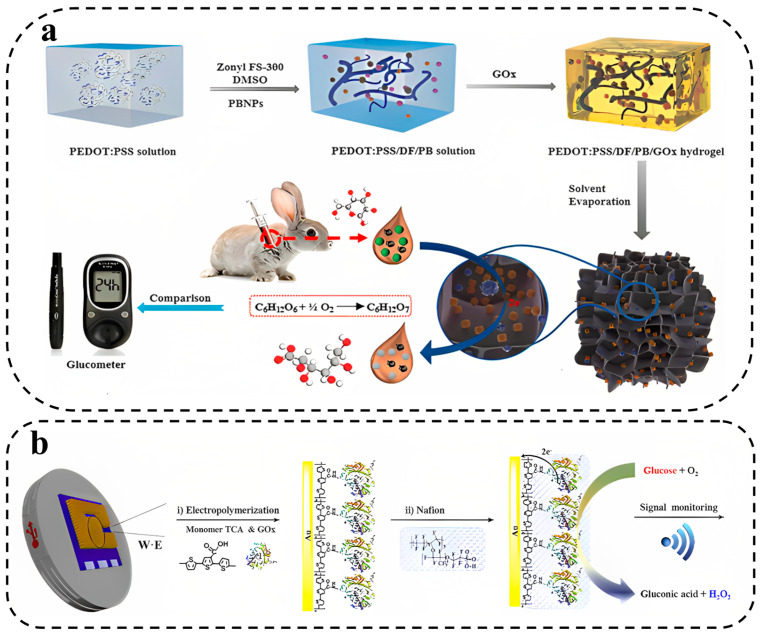
(**a**) PEDOT:PSS hydrogel integrated with Prussian blue nanoparticles for electrochemical glucose biosensing working diagram. Reprinted with permission from Elsevier from ref. [51], and (**b**) The functionalization of the working electrode made of gold with GOx through two steps of electropolymerization and Nafion treatment. Finally, the electrical signals produced during the enzymatic reaction are monitored for measuring the glucose level. Reprinted with permission from Elsevier from ref. [122].

Xu et al. [51] developed a new electrochemical biosensor by using a PEDOT:PSS conductive hydrogel integrated with Prussian blue nanoparticles (PBNPs) that can monitor the glucose on the body PBNPs act as redox species and facilitate electron transfer, which increases the sensitivity of the biosensor. The hydrogel was created using dimethyl sulfoxide (DMSO) and Zonyl FS-300, which allowed for better conductive and nanoporous networks. This hydrogel-based biosensor has a LOD of 0.85 μM and a high sensitivity of 340.1 μA mM^−1^ cm^−2^, which is approximately 10 times greater than agarose and hydroxyethyl methacrylate (HEMA) hydrogels. The authors demonstrate that it is accurate in detecting glucose in serum when compared to a commercial glucometer (Figure 3a). Additionally, hydrogel functionalization with redox species can enable the development of multiplexed biosensors [115].

#### 2.3.2. Biomolecule Species

Biomolecules are naturally occurring molecules that are essential for life processes, such as proteins, carbohydrates, nucleic acids, antibodies and enzymes [116]. Several techniques are used to functionalize hydrogels with biomolecules, including the physical adsorption [117], covalent attachment [118], and encapsulation/bioconjugation [119]. Physical adsorption involves the non-covalent binding of biomolecules to the hydrogel surface [120]. Covalent attachment requires the chemical modification of the hydrogel and the biomolecule to introduce reactive functional groups that can react with each other to form a covalent bond [100]. Covalent attachment can provide a stable and long-lasting attachment of the biomolecule to the hydrogel surface [121]. Another strategy for hydrogel functionalization is the encapsulation/bioconjugation of the biomolecule species into the hydrogel matrix during the synthesis process. The biomolecule can be either physically encapsulated within the hydrogel matrix or chemically cross-linked to the hydrogel network. Encapsulating hydrogels within a protective layer or encapsulation material can provide a shield against moisture loss. This approach helps maintain the hydrogel’s original properties over an extended period [100].

Kim et al. [122] developed a biosensor for glucose monitoring utilizing Gox that was covalently linked to a terthiophene carboxylic acid monomer for functionalization. The functionalized Gox was then electropolymerized on a gold-coated microneedle array using the potential cycling method, resulting in the creation of a sensing probe layer on the needle. To protect the surface and prevent interference from other species, as well as detachment of the probe material, Nafion was applied (as depicted in Figure 3b). The sensor demonstrated a linear response to glucose concentrations ranging from 0.05 to 20.0 mM, exhibiting a sensitivity of 0.22 μA mM^−1^ cm^−2^ and an LOD of 19.4 (±0.62) μA. To validate the reliability of the sensor, it was coupled with a reusable wireless transmitter that transmitted the measured values to a mobile phone via Bluetooth technology.

The biosensor developed by Kim et al. for glucose monitoring utilized Gox, which was covalently bound to a terthiophene carboxylic acid monomer for functionalization and then electropolymerized on a gold-coated microneedle array using the potential cycling method, resulting in the formation of a sensing probe layer on the needle. The surface was protected with Nafion to prevent interfering species and detaching of the probe material (Figure 3b). The sensor exhibited a linear response to glucose concentrations between 0.05 and 20.0 mM with a sensitivity of 0.22 μA mM^−1^ cm^−2^ and an LOD of 19.4 (±0.62) μA. The reliability of the sensor was confirmed through coupling with a reusable wireless transmitter that sends the measured values to a mobile phone via Bluetooth.

## 3. HWEB Properties

### 3.1. Physicochemical Properties

#### 3.1.1. Electrical Conductivity

Conductivity is the distinct advantage of conductive hydrogels, which makes them able to conduct electricity, rendering them highly valuable for a wide range of applications, such as actuating devices, biomedicine, and sensing. Typically, conductive hydrogels consist of cross-linked inherently conductive polymer networks and or a combination of polymer networks and intrinsically conductive materials [123,124]. Their conductivity is primarily attributed to two types of materials, electronic conductive materials (ECMs) and ionic conductive materials (ICMs), as described in Section 2.1.1 and Section 2.1.2.

Conductive hydrogels utilizing ECMs, such as metal nanomaterials, conductive polymers, carbon materials, MXene, etc., often appear black or dark colors, whereas ICMs use ionic conductive electrolytes and polyelectrolytes, which are usually transparent or light in color [125]. Among these conductive materials, CNT was massively studied. For example, by incorporation of CNT into polyacrylamide/polyacrylic acid (PAM/PAA) hydrogels with a conductivity of 0.25 S m^−1^, the conductivity of the composite hydrogel reached 8 S m^−1^ [126]. CNT incorporation into gelatin hydrogels increased the conductivity from 2.5 × 10^−2^ to 7.2 × 10^−2^ S m^−1^ [127]. The conductivity of collagen type I hydrogels was enhanced from 1.4 to 2.4 S m^−1^ by using CNT [128]. CNT also promoted the conductivity of agarose hydrogels from 0.94 to 1.46 S m^−1^ [129]. PEG hydrogel displays a conductivity of 1.14 S m^−1^, which rises to 1.6 S m^−1^ when CNTs are introduced [130]. Other carbon materials, such as GO, can effectively enhance the electrical conductivity of hydrogels. For instance, chitosan hydrogels with a low baseline conductivity of 10^−8^ S m^−1^ exhibited the conductivity of 4 × 10^−2^ S m^−1^ by adding GO into the framework [131]. PU hydrogel exhibits a conductivity of 0.13 S m^−1^ and with the addition of GO and PEDOT:PSS, it reaches 0.62 S m^−1^ [132]. On the other hand, the incorporation of metallic nanoparticles or conductive polymers moderately improves the conductivity of hydrogels. As an example, collagen hydrogel with a very low conductivity of 4 × 10^−7^ S m^−1^ demonstrated a conductivity of 8 × 10^−7^ S m^−1^ with an Ag NPs incorporation [133]. Moreover, the incorporation of PANI into poly(n-vinyl-2-pyrrolidone) (PVP) hydrogels extended its conductivity from 5 × 10^−4^ to 10^−3^ S m^−1^ [134].

Kang et al. [135] introduced a self-powered sensor based on silver-hydrogel (a mixture of acrylic acid, gelatin, N,N′-methylene-bis(acrylamide), 2-hydroxy-4′-(2-hydroxyethoxy)-2-methylpropiophenone in water and glycerin)/polydimethylsiloxane (Ag-Hydrogel/PDMS) composite film. Researchers reported that the conductivity of Ag-Hydrogel/PDMS increased significantly with an increase in the amount of Ag nanocubes (Ag NCs), but after a specific concentration, conductivity gradually increased, suggesting that the concentration of Ag NCs reached a critical level. Another member of ECMs is conductive polymers, which possess a distinctive π-conjugated structure, enabling them to accomplish electron transfer [136]. Peng et al. [137] prepared a conductive hydrogel by in situ polymerization of conductive PEDOT in PVA aqueous solution for the strain-sensitive application, which showed a good conductivity of ~0.95 S·m^−1^. CNMs can be used to prepare electron-conductive hydrogels due to their high electrical conductivity. A common approach for designing these materials involves dispersing them into an aqueous phase of hydrogel before cross-linking the components to form conductive networks [136]. However, recent studies showed that new promising materials, such as MXenes, are ideal for the preparation of conductive hydrogels [138]. Liu et al. [66] indicated that the hydrophilic Ti_3_C_2_ MXene enhances the rheology properties that make it possible to create an extrusion printing ink that is fully aqueous and improves the conductivity of aqueous PEDOT:PSS inks for 3D printing of hydrogel. The 3D-printed hydrogel with a water content of 96.6 wt.% has an unprecedented conductivity of 15.25 S cm^−1^.

When it comes to ICMs, hydrogels typically have 3D network structures and continuous liquid phase, providing numerous channels for ion migration, which is essential for ICMs [139]. By directly dissolving electrolytes with abundant cation and anion carriers in hydrogel precursors, hydrogels are effectively endowed with excellent conductivity. The concentration of doped ions determines the conductivity and sensitivity of hydrogels in the case of wearable sensors [140]. For instance, the incorporation of NaCl into polymer networks of allyl cellulose led to a conductivity of 1.8 × 10^−5^ S m^−1^, while for PAM/PDA with NaCl and PDA in the polymer networks, the conductivity reached 0.27 S m^−1^ [125]. Yue et al. [141] developed a hybrid composite hydrogel by incorporating dialdehyde micro-fibrillated cellulose (DAMFC) fibrils and chitin nanowhiskers (ChNs) synergistically enhanced by gelatin (referred to as DAMFC/ChN/gel). The hydrogel exhibited high ionic conductivity even at low temperatures, such as −20 °C, due to its soaking in NaCl solution. This is attributed to the presence of ions in the hydrogel, which lowers the freezing point of water and enables efficient ion conductivity. As water molecules tend to crystallize at low temperatures, the hydrogel’s incorporation of ions helps maintain its ionic conductivity under such conditions.

In general, electronic conductive hydrogels tend to exhibit higher conductivity compared to ionic conductive hydrogels. However, their cost can pose a barrier to their widespread use. Furthermore, electrochemical sensors are reliant on electrical signals, underscoring the importance of hydrogels possessing a degree of conductivity. A higher level of conductivity in the hydrogels translates to better electrochemical signals [142]. It is important to note that the incorporation of electronic conductive materials into hydrogels may negatively impact their mechanical properties by interfering with the formation of the polymer network. On the other hand, the presence of ions in electrolytes or polyelectrolytes tends to enhance the mechanical properties of hydrogels through coordination interactions with the polymer or the salting out effect [125].

#### 3.1.2. Diffusibility

One of the key characteristics of hydrogels is their diffusibility, which refers to the ability of molecules to diffuse through a hydrogel matrix [143]. To achieve accurate detection in HWEBs, target molecules must diffuse easily through the hydrogel matrix and reach electrode surfaces [144]. Low diffusion is a major limitation of wearable sensing platforms used to detect samples, such as organophosphorus, fentanyl, and drug powders [145].

Several factors can affect the diffusibility of molecules in hydrogels, including the size, shape, and charge of molecules, as well as the porosity and cross-link density of hydrogels. For instance, small molecules can easily diffuse through the hydrogel matrix, while larger molecules in weight and size may diffuse in a slower manner [146]. Additionally, the shape of the molecule can influence its diffusibility. Linear molecules may diffuse more easily than bulky or branched molecules [147]. The charge of the molecules can also influence the diffusibility of hydrogels. Charged molecules may interact with charged polymer chains in a hydrogel matrix, resulting in electrostatic repulsion or attraction. This can either enhance or hinder the diffusion of charged molecules within the hydrogel network [148]. The porosity of a hydrogel matrix also plays a crucial role in hydrogel diffusibility. Highly porous hydrogels with large pore sizes allow for easy diffusion of molecules, while dense hydrogels with small pores may restrict molecular diffusion [143]. Furthermore, the cross-link density of a hydrogel matrix can affect diffusibility. Cross-linking between polymer chains restricts the mobility of the chains, which may hinder the diffusion of molecules [143]. In this respect, Abraham et al. [149] manipulated the mesh size of a thermoresponsive double network nano-composite hydrogel (DNNC) composed of poly(N-isopropylacrylamide) (PNIPAAm) and polysiloxane nanoparticles. They achieved this by adjusting factors such as crosslink density and monomer concentration. To assess the mesh size, the researchers conducted a size exclusion experiment using FITC-dextran molecules with varying sizes (4, 10, 20, 40, 70, 150, and 250 kDa MW). The results revealed that the DNNC hydrogels had a mesh size ranging from 6.5 to 9.6 nm at room temperature, which corresponds to hydrodynamic diameters (Dh) of 20 kDa and 40 kDa FITC-dextran, respectively. Consequently, the glucose molecule (with a Dh of approximately 1 nm) would be able to diffuse freely through this membrane. Therefore, the biosensor membrane would not significantly impede the diffusion of the analyte.

Overall, diffusibility is an essential property of hydrogels that can influence HWEBs’ performance, and its optimization should not adversely affect its other properties, especially their mechanical properties.

#### 3.1.3. Hydrophilicity

Hydrophilicity is a crucial property of hydrogels determining their behavior in contact with aqueous solutions. Hydrophilic hydrogels prevent sensor biofouling by binding with the water on their surfaces rather than microorganisms in the surrounding environment [150]. Hydrophobic hydrogels are less common than hydrophilic hydrogels, but in terms of wearable biosensors, hydrophobicity can also be useful in creating a barrier between the sensor and the surrounding environment, preventing interference from other biomolecules [151]. In a study conducted by Zeng et al. [152], the impact of hydrophobicity on the anti-fouling properties of various hydrogels with different water content and chemical structures was compared. The researchers observed that hydrophobic hydrogels exhibited notable surface hydrophobicity, as evidenced by high static water contact angles (WCAs) exceeding 90°. This characteristic is uncommon for traditional hydrogels. Specifically, hydrogels composed of poly(2-(2-ethoxyethoxy)ethyl acrylate) (PCBA) and poly(tetrahydrofurfuryl acrylate) (PTHFA) displayed high surface hydrophobicity. Remarkably, even after being incubated in a bacterial suspension for 7 days, these hydrogels demonstrated only 5.1 and 2.4% coverage of E. coli biofilm, respectively. This was significantly lower compared to the hydrophilic poly(N,N-dimethylacrylamide) (PDMA) hydrogels, which exhibited biofilm coverage approximately 0.32 and 0.15 times higher, respectively. The research findings indicate that the effectiveness of hydrophobic hydrogels in preventing fouling is primarily attributed to their surface hydrophobicity.

The chemical structure of polymer chains in hydrogels plays a significant role in determining their hydrophilicity or hydrophobicity. For example, poly(acrylic acid) (PAAc), a widely used hydrogel-forming polymer, contains carboxylic acid groups that can form hydrogen bonds with water molecules, making it highly hydrophilic, also hydrophobic hydrogels reduce the attachment between hydrogel surface and fouling agents [153]. The degree of cross-linking in hydrogels also affects their hydrophilicity or hydrophobicity. Highly cross-linked hydrogels tend to be less hydrophilic than loosely cross-linked hydrogels due to reduced water accessibility to polymer chains [154]. Additionally, the type of cross-linker and pH of the environment can influence the hydrophilicity of hydrogels [155]. The properties of HWEBs can be tuned to optimize their performance, and the selection of hydrophilic/hydrophobic properties of hydrogels must be carefully balanced with the mechanical requirements of HWEBs.

#### 3.1.4. Self-Healing Property

The self-healing ability allows hydrogels to repair their structure after being damaged, increasing their lifespan and reliability. Self-healing behavior can be induced through either external stimuli or autonomous interactions within the hydrogels [156]. In the first approach, external stimuli, such as heating or adding a self-healing agent, are required to trigger the self-healing ability in the hydrogels [156]. In an illustrative instance, supramolecular networks were created by combining crystalline polyethylene glycol (PEG) and poly(ε-caprolactone) through the utilization of 2-ureido-4-pyrimidone supramolecular moieties. This configuration allowed for the conversion of light into heat, resulting in polymer films being heated to a temperature of 63 ℃ when subjected to UV irradiation. Consequently, this process triggers the activation of a temperature-dependent self-healing mechanism [157].

On the other hand, the self-healing mechanism that operates independently without the need for external stimuli can be classified into two types. Firstly, there is the category of dynamic chemical bonding, encompassing an acylhydrazone bond, a Schiff-base bond, a disulfide bond, a boronate ester bond, and Diels–Alder reactions. Secondly, there is the category of non-covalent bonding, which includes metal coordination, hydrophobic interaction, π–π stacking, ionic interaction, host–guest interactions, and hydrogen bonds [158].

**Figure 4:**
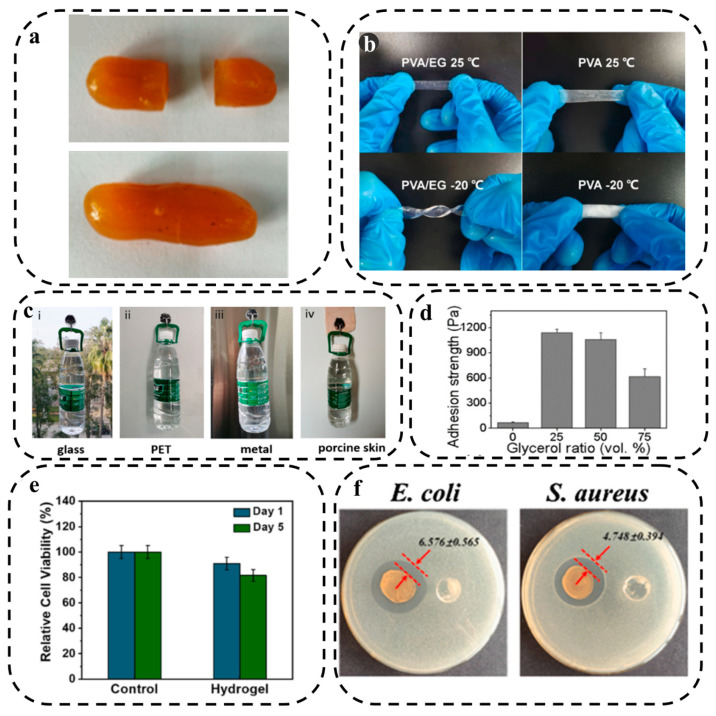
(**a**) The self-healing ability of the DN hydrogel. Reprinted with permission from ACS from ref. [159], (**b**) Comparing 25%PVA/EG hydrogel with PVA hydrogel at 25 °C and −20 °C. Reprinted with permission from Elsevier from ref. [160], (**c**) MXene/PMN hydrogels can adhere to a variety of surfaces, including (i) glass, (ii) PET, (iii) metal, and (iv) porcine skin. Reprinted with permission from Elsevier from ref. [161], (**d**) Porcine skin adhesion to G-P-C@agarose gels with different glycerol contents. Reprinted with permission from ACS from ref. [162], (**e**) Cell viability of NIH3T3 cells on Day 1 and Day 5, cultured with PDDA/CNF hydrogels and the control group. Reprinted with permission from Elsevier from ref. [163], and (**f**) Observation of the inhibition zone on the culture dishes for control and PNAg-hydrogel samples relative to *E. coli* and *S. aureus*. Reprinted with permission from Elsevier from ref. [164].

Liu et al. [159] developed multifunctional double network (DN) hydrogels that demonstrated a remarkable healing capability, achieving a healing efficiency of 95%. The researchers achieved self-healing properties in the hydrogel by employing the host-guest interaction between β-cyclodextrin (as the host) and ferrocene (as the guest), utilizing the dynamic borate ester bonds present in the poly(vinyl alcohol) and borax components (Figure 4a).

#### 3.1.5. Anti-Freezing Property

It is crucial for a hydrogel-based biosensor to tolerate a wide range of environmental temperatures. Traditional hydrogels use pure water as a dispersion medium, which can freeze easily at low temperatures or evaporate quickly at high temperatures, resulting in rigid, fragile hydrogels that limit the ionic conductivity of the hydrogels [165]. To address this problem, two main strategies have been developed for preparing HWEBs, including the addition of cryoprotectants and the modification of polymer networks. Cryoprotectants can depress water icing in hydrogels in two colligative ways, i.e., (I) replacing the aqueous solution with a mixture of water and cryoprotectant, such as ethylene glycol (EG), glycerol, sorbitol, etc. and (II) adding salt [166]. A mixture of EG and water has a freezing point below −40 °C because EG and water form hydrogen bonds that reduce the amount of free water in the hydrogel matrix and disrupt ice crystal formation. The hydrogen bonds between water and EG can also prevent water evaporation [166]. For example, a PVA/EG conductive hydrogel was constructed by replacing a pure water solvent with an EG/water mixed solvent, which showed excellent anti-freezing and moisturizing properties at both −20 and 25 °C (Figure 4b) [160].

Inorganic salts can also effectively depress the formation of ice crystals due to their colligative properties [167]. Zeng and colleagues [168] developed a hydrogel suitable for wearable sensors by grafting acrylamide and acrylonitrile copolymers onto cellulose chains in the presence of zinc chloride. The initiator used in this process was ceric ammonium nitrate. The presence of zinc chloride was found to have an impact on the antifreeze performance of the hydrogel, enabling it to remain soft and flexible even at −20 °C. In contrast, the hydrogel without zinc ions (Zn^2+^) became opaque and rigid at the same low temperature, losing its ability to bend.

In addition, an effective approach to address the freezing issue in hydrogels is through the modification of polymer networks. This strategy involves altering the structure of the polymer network to ensure that unfrozen water primarily consists of water molecules that are tightly associated with the polymer network [169]. Although this method provides a new perspective on anti-freezing hydrogels, it is still uncommon for the preparation of wearable devices [166].

#### 3.1.6. Adhesion

To increase the repeatability and accuracy of detection, especially subtle physiological changes, it is important for wearable biosensors to adhere to the human body without the use of any additional support, such as adhesive tapes or bandages [166]. Hydrogels are able to integrate with human skin without the need for additional adhesives and can be easily removed without causing any harm to the skin [170]. Usually, adhesion occurs due to a chemical or physical interaction between disparate surfaces at soft and hydrated interfaces. Hydrogels have low densities of functional groups and are wet and deformable, making it difficult to form these adhesion junctions [171].

Chen et al. [161] developed an MXene/polyampholytes (PMN) nanocomposite hydrogel with abundant dynamic bonds, including ionic and hydrogen bonds, which can adhere to different surfaces. The MXene/PMN hydrogel was coated onto a hook and successfully adhered to glass, PET, metal, and porcine skin while it bore a bottle of water (Figure 4c). Han et al. [162] added glycerol to a polydopamine (PDA)-CNTs@agarose (G–P–C@agarose) gel to increase its adhesiveness. P–C@agarose gels exhibited weak adhesion with a value of approximately 67 ± 8 Pa, attributed to the properties of PDA, whereas the adhesion strength of G–P–C@agarose gels significantly improved nearly 17 times by adding 25 vol% glycerol. However, adding glycerol to the composite over 25 vol% decreased the adhesion strength as the gel and porcine skin may slip on each other (Figure 4d).

### 3.2. Biological Properties

The biological properties of hydrogels play a crucial role in determining their potential in various biomedical applications, such as HWEBs. In this part, we will focus on hydrogel biocompatibility and antibacterial properties.

#### 3.2.1. Biocompatibility

Wearable biosensors need to be biocompatible when in contact with the human body to prevent inflammation and other serious health issues [172]. Biocompatibility is defined as the ability of a biomaterial to perform its function without causing toxic or injurious effects to biological systems, as long as it has the ability to induce an appropriate host response based on the specific application [173]. Hydrogels are a promising option for meeting this requirement. However, some hydrogels lack biocompatibility and can be toxic to the body [174,175]. Toxic chemicals, such as many crosslinking agents in synthetic hydrogels, can be an obstacle to achieving biocompatibility in hydrogels [176].

Biocompatibility can be assessed by identifying the relative number of live and dead cells within the polymer matrix [177]. Wei et al. [163] fabricated a double network composite hydrogel, namely PDDA/CNF, by incorporating dopamine methacrylamide (DMA), methacrylatoethyl trimethyl ammonium chloride (DMC), acrylic acid, and cellulose nanofibers (CNF) as the filler. The researchers conducted a cell viability assessment by culturing NIH 3T3 cells on the PDDA/CNF hydrogels and employing the cell counting Kit-8 (CCK-8) assay after 1 and 5 days (Figure 4e). The results demonstrated excellent biocompatibility of the hydrogel as the cells exhibited strong adhesion and maintained a healthy state, with a majority of cells displaying green staining and a well-spread morphology in both experimental groups. Moreover, the researchers quantitatively determined the relative survival rate of the cells, revealing a survival rate of 90.9% after 1 day and 81.6% after 5 days. These findings exceed the standard requirement for cytotoxicity (70.0%) by a significant margin.

#### 3.2.2. Antibacterial Property

The high-water content of hydrogels makes them prone to absorbing microorganisms, potentially leading to infections [178]. To address this issue, antibacterial hydrogels are being developed for HWEBs to inhibit bacterial infections. This property can prevent allergic symptoms of the skin directly in contact with HWEBs and, moreover, can prolong the service life of the HWEBs and reduce their need for replacement [179].

The antibacterial property can be evaluated using the bacteriostatic zone method. This involves testing the bacteriostatic zone diagram and diameter of the hydrogel compared to typical Gram-negative bacteria, such as Escherichia coli and specific Gram-positive bacteria, like Staphylococcus aureus in an in vitro antibacterial experiment [180,181].

Several bioactive antibacterial materials have been introduced, encompassing a diverse range of options. These include metallic-based nanomaterials, such as silver, zinc, gold, and copper. Additionally, there are photothermal antibacterial agents like polydopamine (PDA), CNTs, and GO, as well as photodynamic antibacterial agents like molybdenum disulfide (MoS_2_) and titanium dioxide (TiO_2_). Antibacterial polymers, such as chitosan, polyethyleneimine, and 3-polylysine, have also demonstrated their efficacy. Furthermore, extracts derived from natural products, such as honey and essential oils, have shown antibacterial properties [92]. To create an antibacterial hydrogel-based nanocomposite sensor named PNAg, silver nanoparticles-doped cellulose nanofibers (AgNPs-doped PDA@NFC) were incorporated into a covalently cross-linked polyacrylamide (PAm) network. The antibacterial activity of the PNAg nanocomposite hydrogel was evaluated using the inhibition zone test against *Escherichia coli* (*E. coli*) and *Staphylococcus aureus* (*S. aureus*). After 24 h, the control hydrogels did not exhibit any antibacterial rings, whereas the PNAg hydrogel demonstrated a 6.576 mm inhibition zone for E. coli and a 4.748 mm inhibition zone for S. aureus. These results clearly indicate the effective antibacterial property of the PNAg hydrogel (Figure 4f) [164].

#### 3.2.3. Biodegradability

It is undeniable that the environment is being increasingly polluted by non-degradable materials, which require proper disposal. To address this issue, biodegradable materials are a viable solution, as they can be broken down by humidity and microorganisms in the environment [182,183]. The biodegradability of hydrogels is determined by the components present in the system and the preparation methods employed. Degradation typically involves the hydrolysis and solubilization of biological entities within the hydrogel [21]. The development of biodegradable hydrogels for wearable biosensors has garnered significant attention in recent years. For instance, Lu et al. [184] has described a biodegradable sensor comprising bacterial cellulose (BC) hydrogels as the matrix and imidazolium perchlorate (ImClO_4_) molecular ferroelectric as the functional element. According to their study, the BC hydrogel can be completely degraded into glucose and oligosaccharides, while ImClO_4_ can be recycled and reused in the same devices, resulting in zero environmental pollution. In another study, poly(thioctic acid) was used as the skeleton structure and mixed with polydopamine to create a multifunctional hydrogel that gradually became smaller over time and was almost entirely degraded under the skin after 18 days [185].

### 3.3. Rheological and Mechanical Behavior

The rheological and mechanical behavior of hydrogels plays a crucial role in the robustness and durability of HWEBs, as the device undergoes external forces like stretching, compression, bending, and twisting during monitoring. This section will discuss the mechanical properties of hydrogels, such as stretchability, swelling behavior, and cycle life, as well as their rheological behavior, which are essential factors to be considered in terms of the final application.

#### 3.3.1. Stretchability

Achieving proper function during limb activities is crucial for wearable electronic devices, and stretchability is an essential factor in achieving this goal [186]. Stretch is measured by dividing the length of the hydrogel in a deformed state by the length of the hydrogel in the initial undeformed state [187]. Stable compliance between HWEBs and sensing substrates which will be achieved by effective stretchability, not only allows the devices to acquire noise-free responses but also enhances the sensitivity and LOD of the biosensors [188].

Hydrogels offer excellent stretchability and weak but adjustable mechanical properties (for example, tensile strength, toughness, etc.), making them promising candidates for HWEBs. By adjusting the type and density of cross-linking, their mechanical strength can be optimized. It may be possible to strengthen a hydrogel by increasing the cross-linking degree; while a greater degree of cross-linking results in the formation of a stronger hydrogel, it reduces the stretchability, leading them to be brittle. Therefore, optimum cross-linking should be considered for relatively robust and elastic hydrogels [189].

**Figure 5:**
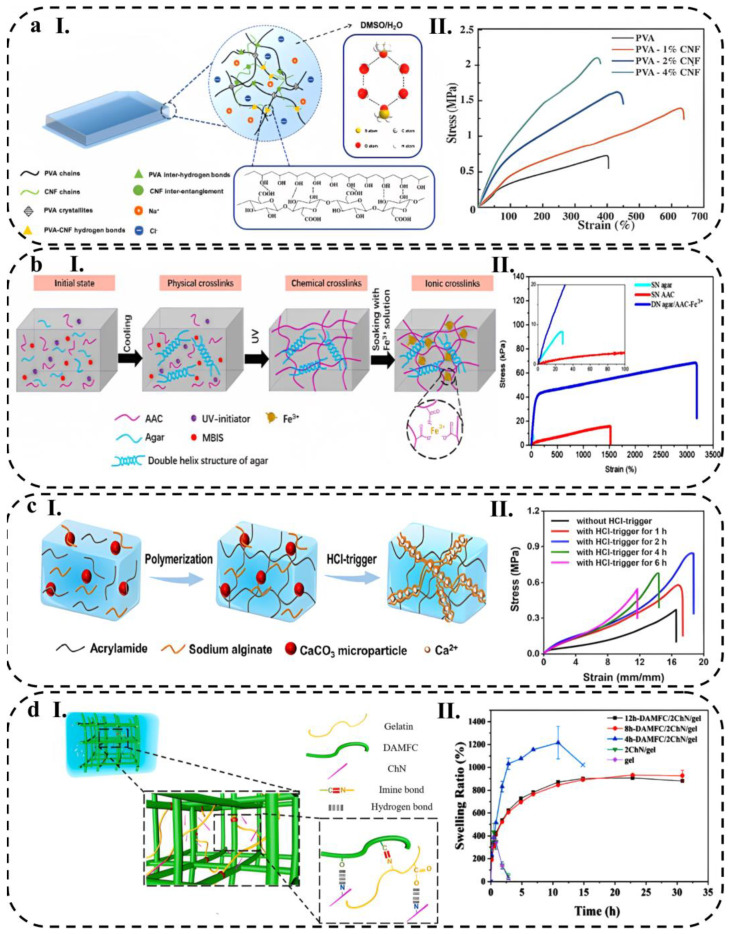
(**aI**) Schematic illustration of PVA–CNF organohydrogel, (**aII**) Tensile stress–strain curves for PVA–CNF organohydrogels with varying amounts of CNF. Reprinted with permission from Wiley from ref. [193], (**bI**) Schematic illustration of the DN agar/AAc–Fe^3+^ hydrogel, (**bII**) The comparison of tensile stress–strain curves between the SN agar, SN AAc, and DN agar/AAc–Fe^3+^ hydrogels. Reprinted with permission from ACS from ref. [196], (**cI**) The formation of Ca^2+^/SA/PAM DN hydrogels using homogeneous Ca^2+^ cross-linking strategies, (**cII**) Tensile stress–strain curves of Ca^2+^/SA/PAM DN hydrogels at different incubation times in HCl. Reprinted with permission from RSC from ref. [197], (**dI**) Formation mechanism of DAMFC/ChN/gel hybrid composite hydrogel, and (**dII**) Swelling behavior of lyophilized DAMFC/ChN/gel hybrid composite hydrogels. Reprinted with permission from Elsevier from ref. [141].

In recent years, many studies have been conducted to improve hydrogel stretchability by incorporating nano- and micro-scale reinforcements [190,191,192]. By adding an optimal amount of fillers to hydrogels, abundant chemical or physical bonds can be formed between polymer chains and functional groups of fillers, thus improving the hydrogel’s stretchability and mechanical strength [170]. For instance, Ye et al. [193] added cellulose nanofibrils (CNF) to PVA organohydrogel for mechanical reinforcement (Figure 5(aI)). The addition of 1% wt. of CNF content increased the stretchability and ultimate tensile stress of pure PVA organohydrogel from 400 to 660% and 0.6 to 1.4 MPa, respectively. CNFs play a crucial role in reinforcing the organohydrogel and dissipating energy during deformation by forming hydrogen bonds with PVA chains and/or physical entanglement with themselves. However, increasing the CNF content over 2%, which is typical for composites reliant on nanofillers, can decrease the elongation at break value (i.e., stretchability) (Figure 5(aII)).

The shape of fillers can also affect the stretchability of hydrogels. In this context, Xia et al. [194] compared the effect of various particle shapes, including three-dimension materials, such as kaolin (KL-3D) and montmorillonite (MMT-3D), two-dimension material of rGO (rGO-2D), and one-dimension material of attapulgite (APT-1D), on the stretchability of PAM hydrogels. The results showed that rGO exhibited a higher stretchability due to preventing excessive cross-linking of hydrogels during polymerization and maintaining the original polymer matrix structure. As rGO can deflect and rotate along with the deformation of the hydrogel, it acts as an intermolecular lubricant, greatly improving the tensile fracture properties of hydrogel with a tensile strain of 568%.

Double-network (DN) hydrogels are a type of hydrogel that possess proper stretchability and simultaneously suitable mechanical strength. Due to the rigidity, brittleness, and tight cross-linking of the first network, DN hydrogels dissipate a significant amount of energy during deformation, acting as sacrificial bonds. In contrast, the second network is usually soft, ductile, and loosely cross-linked, acting as a hidden length that protects the integration of DN hydrogel when the first network fractures [195]. Li et al. [196] introduced an agar/acrylic acid (AAc) hydrogel with a unique structure consisting of a brittle network formed by physically cross-linked agar and a ductile network created through chemical cross-linking of AAc. In their study, they incorporated a coordination interaction between Fe^3+^ ions and carboxylic acid groups present in AAc to enhance the mechanical and electrical properties of the hydrogels (Figure 5(bI)). The resulting double network (DN) hydrogel exhibited remarkable stretchability, with an elongation at the break reaching 3174.3%. This exceptional stretchability was attributed to the reversible ionic interaction between AAc and Fe^3+^, which facilitated the unfolding and refolding of AAc chains (Figure 5(bII)).

In a separate study, a homogeneous DN hydrogel composed of calcium ion (Ca^2+^), sodium alginate (SA), and polyacrylamide (PAm) was developed using a controlled acid-triggered release of Ca^2+^ for cross-linking SA (Figure 5(cI)). The acid-triggered cross-linking process generated sacrificial ionic bonds within the hydrogel, enabling energy dissipation and resulting in notable improvements in stretchability (1850%) and tensile strength (0.85 MPa) of the Ca^2+^/SA/PAm DN hydrogel (Figure 5(cII)) [197].

Hydrogel tensile properties, such as stretchability and tensile strength, can also be improved by incorporating metal ions into hydrogel matrices. These properties are influenced by two factors related to the metal ions, (I) their coordination ability and (II) their electrostatic bonding strength and concentration [198,199]. Zang et al. [200] developed a polyacrylamide/copper-alginate (alg) DN hydrogel with a tensile strength of 2.25 ± 0.02 MPa. The ionic interaction between alg and Cu^2+^ can dissipate a large amount of energy when the PAM/Cu-alg DN hydrogel is stretched. Additionally, the hydrogen bonds between PAM chains can also dissipate energy, enhancing the hydrogel’s mechanical strength (Table 1).

It is worth noting that there are other construction strategies for improving the stretchability of hydrogels in HWEBs, such as slide-ring hydrogel (topological) [201], macromolecular microsphere composite hydrogel [202], and supramolecular hydrogels [203]. However, it can be concluded that cross-linking density is the main factor in optimizing and balancing the mechanical and stretchability features of the hydrogels [204].

**Table 1:** Mechanical properties and features of wearable strain sensors.

Material (s)	Mechanical Performance			Strain Sensing				Features	Ref
	Tensile Strength (MPa)	Stretchability (%)	Toughness (MJ m^−3^)	Gauge Factor (Strain Range)	Conductivity (S cm^−1^)	Conductive Type	Repeatability (Cycles)		
PVA/NaCl/Amy ^(1)^		-	0.184	4.96	0.034	Ion	100	Biocompatible Anti-swell Anti-fatigue	[205]
PVA/P(AAc- co-AM)/PDA@CNTs ^(2)^	~1.21	~220	~1.22	1.6	3.84	Electron	-	Fatigue resistant Recoverable	[192]
P(AAc-MEA)-Fe^3+ (3)^	0.462	~1200	2.01	1.60 (0–100%), 1.97 (100–200%), 2.57 (200–400%)	0.044	Ion	-	Anti-swelling Recoverable	[206]
CNTs/PVA ^(4)^	-	up to 415	-	0.591 (0–150%), 1.165 (150–250%)	1.11	Electron	1000	Durable	[191]
PANi/CBH ^(5)^	0.8	~914	4.3	0.5 (0–90%), 1.7 (90–600%)	0.3	Electron	300	Biocompatible Skin mimicking Self-stiffness	[207]
PVA-CNF ^(6)^	1.4	up to 660	5.25	1.2 (<150%), 1.5 (>150%)	3.2	Ion	500	Anti-freeze Long-term solvent retention	[193]
PAM/Cu-alg ^(7)^	~2.25	2013	-	up to 5.1	0.408	Ion	-	-	[200]
PATG-B-Fe^3+ (8)^	0.203	1950	-	1.2 (0–400%), 3.3 (400–1200%) 5.2 (1200–1900%)	0.237	Ion	2000	Self-heal Self-adhesive	[208]
MCNH ^(9)^	0.0145	up to 2000	-	0.83	9.43	Ion	-	Self-healing	[209]
PVA/LNP/AlCl_3_ ^(10)^	1.241	589	-	2.08	1.35 × 10^−2^	Ion	-	Anti-freezing	[190]
CNT/TPU ^(11)^	73.22	476	-	11.08	0.023	Electron	1250	-	[210]
PAC-CGO-Na ^(11)^	1.51	1414	15.33	4.44 (220–1216%)	4.10	Ion	200	Anti-freezing	[211]
PVA/gelatin/EG/TA@CNC–Al^3+^ (PGETA) ^(12)^	1.95	~520	-	4.23	0.23	Ion	1000	Self-heal Recyclable	[212]
poly(ACMO)/glycerin/PEGDA ^(13)^	0.18	~356	-	2.3	1.9 × 10^−3^	Ion	700	Self-adhesive Fatigue resistant	[213]
PAAc/glycerin/PVA/PEDOT ^(14)^	3.6	340	-	1.18 (0–400%)	~0.95	Electron	-	Anti-freeze	[137]
PANi/P(AAm-co-HEMA) ^(15)^	7.27 (72 h oxidation)	530	9.19	11 (at low strain)	8.24	Electron	100	Fatigue resistant	[214]
PVA/glycerol/PANi ^(16)^	0.094	472	-	2.14	0.32	Electron	540	Anti-freeze Remoldable Reusable	[215]
SC/PDA/PAAm ^(17)^	0.170	over 2100	1.1	-	-	Ion	-	Self-heal Self-adhesive	[216]
HP(AAm/AAc)–CS–Fe^3+ (18)^	4.05	-	23.8	3.621	-	Ion	300	Anti-swell Recoverable	[217]

^(1)^—Polyvinyl alcohol/sodium chloride/amylopectin. ^(2)^—Polyvinyl alcohol/poly(acrylic acid-co-acrylamide)/polydopamine-decorated carbon nanotubes. ^(3)^—Chitosan (CS)/poly(acrylic acid-2-methoxyethyl acrylate)-Fe^3+^. ^(4)^—Carbon nanotubes/polyvinyl alcohol. ^(5)^—Polyaniline/cellulose biomimetic hydrogel. ^(6)^—Polyvinyl alcohol-cellulose nanofibrils. ^(7)^—Polyacrylamide/copper-alginate. ^(8)^—Bacterial cellulose nanowhisker (BCW), tannic acid (TA), polyacrylic acid (PAAc), Fe^3+^ and glycerol/water (Gly/H_2_O). ^(9)^—Multiple cross-linked network hydrogel (acrylic acid (AAc), stearyl methacrylate (SMA), sodium dodecyl sulfate (SDS, N,N’-methylenebisacrylamide (MBAAm), and CaCl_2_. ^(10)^—Carbon nanotube/thermoplastic polyurethane. ^(11)^—Polyacrylamide/chitosan modified graphene oxide/sodium. ^(12)^—Tannic acid-coated cellulose nanocrystals/polyvinyl alcohol/gelatin/ethylene glycol/Al^3+^. ^(13)^—Acrylic-, N-acryloylmorpholine/glucerine/poly(ethylene glycol) diacrylate. ^(14)^—Polyacrylic acid/glycerin/polyvinyl alcohol/poly(3,4-ethylenedioxythiophene). ^(15)^—Polyaniline/poly(acrylamide-co-hydroxyethyl methylacrylate). ^(16)^—Polyvinyl alcohol/glycerol/polyaniline. ^(17)^—Sodium casein/polydopamine/polyacrylamide. ^(18)^—Hexadecyl methacrylate/acrylamide/acrylic acid/chitosan/iron ions.

#### 3.3.2. Swelling Behavior

Hydrogels have a significant characteristic of absorbing water or thermodynamically compatible solvents, which is known as swelling and is expressed by the swelling ratio. The swelling of hydrogels involves three steps, diffusion of water into the hydrogel network, loosening of polymer chains, and expansion of the hydrogel network [21]. This ability originates from polar and hydrophilic groups of hydrogels, such as −SO_3_H, −OH, −NH_2_, −COOH, −CONH_2_, and many more [21]. Several factors, such as the hydrophilicity of functional groups, swelling medium, and strength of crosslinked bonds, impact the swelling degree. Of all these factors, the strength of crosslinking plays a more crucial role in both the extent of water absorption and the maintenance of the swollen structure [21]. The extent of swelling in hydrogels is determined by the competition between the osmotic diffusion of the solvent and the force exerted by the polymer cross-linking network [218]. The swelling ratio is typically calculated by taking the difference between the weights of the swollen ($W_{swollen}$) and dried ($W_{dry}$) hydrogels after normalizing to the weight of the dried hydrogel [219]:

(1) $$Swelling\;ratio=\frac{W_{swollen}-W_{dry}}{W_{dry}}$$

The hydrogels are commonly swollen in phosphate-buffered saline (PBS). The swelling behavior is affected by various factors, such as the nature of the polymer, the degree of crosslinking, functional groups in polymer chains, ionic media, synthesis state, and pH of the medium [21]. Controllable swelling is an important property of HWEBs. The analyte diffusion time in HWEBs can be directly related to the swelling kinetics of hydrogel, emphasizing the importance of assessing the swelling behavior of hydrogels in the field of wearable biosensors.

Yue et al. [141] synthesized hybrid hydrogels using chitin nanowhiskers (ChN) and dialdehyde microfibrillated cellulose (DAMFC) fibrils, which were synergistically enhanced by gelatin (Figure 5(dI)). The incorporation of dialdehyde cellulose and the utilization of chemical crosslinking contributed to an improved swelling ratio compared to pristine gelatin. However, it was observed that the equilibrium swelling ratio of the DAMFC/ChN/gel hybrid composite hydrogels decreased as the cellulose oxidation time increased (Figure 5(dII)). Despite this decrease, the hydrogels demonstrated excellent stability and maintained their integrated shapes when immersed in PBS for over 30 h. The reduction in swelling ratio and the ability to retain shape were attributed to the formation of a denser network structure within the hydrogel system.

**Figure 6:**
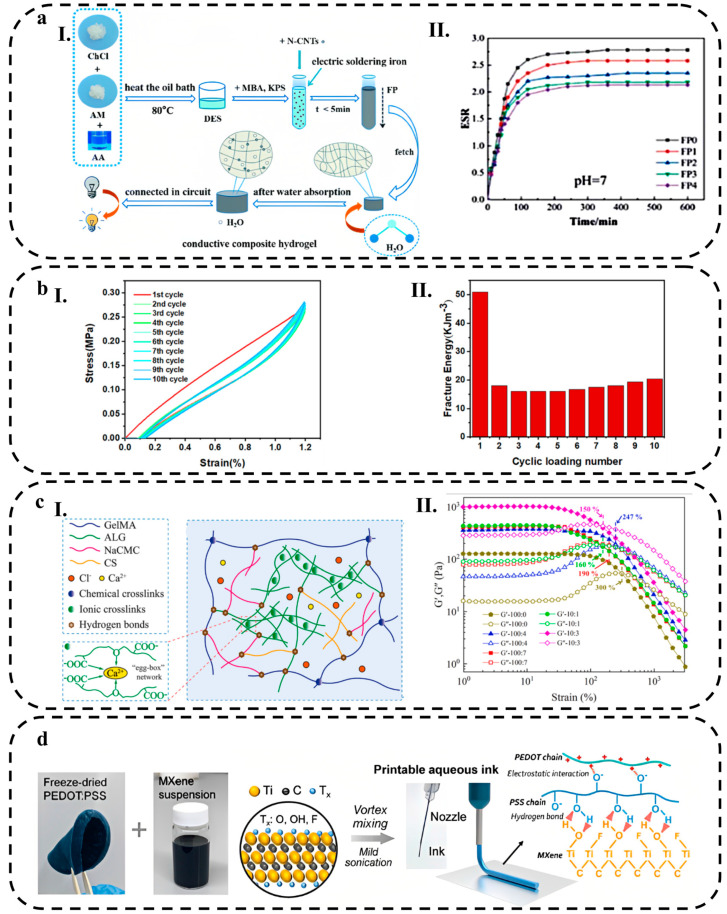
(**aI**) Schematic diagram of the preparation, (**aII**) The swelling kinetics curve of the N-CNTs/P(AAc–co–AAm) composite hydrogel at pH = 7. Reprinted with permission from RSC from ref. [76], (**bI**) Loading–unloading curve of PVA/P(AAc–co–AAm)/PDA@CNTs composite hydrogel after ten cycles of 120% strain stretching, (**bII**) The amount of energy that dissipated from ten cycles stretching of the composite hydrogel at 120% tensile strain. Reprinted with permission from Nature from ref. [192], (**cI**) The composite hydrogel’s proposed molecular structure, (**cII**) The comparison of the storage modulus (G′) and loss modulus (G″) of hydrogels with different mass ratios at 25 °C. Reprinted with permission from Elsevier from ref. [220], and (**d**) Schematic illustration of the preparation of ink for extrusion printing prepared from PEDOT:PSS functionalized with MXene. Reprinted with permission from Wiley from ref. [66].

In a different investigation, the swelling behavior of a composite hydrogel composed of acrylic acid (AAc), acrylamide (AAm), choline chloride (ChCl), and nitrogen-doped carbon nanotubes (N–CNTs) was studied (Figure 6(aI)). The inclusion of N–CNTs in the hydrogel, ranging from 0 wt.% in FP0 to 1 wt.% in FP4, led to a physical entanglement of chain macromolecules, thereby increasing the cross-linking density and reducing the swelling properties of the hydrogel (Figure 6(aII)). Furthermore, the impact of pH on the swelling behavior of the composite hydrogels was examined. Under strongly acidic conditions, the hydrogen bond interactions between carboxylic acid groups (−COOH) and the polymer chain caused entanglement and contraction of the polymer chain throughout the network, resulting in a low swelling ratio. Conversely, under alkaline conditions, the carboxylic acid groups dissociated into carboxylate ions (−COO−) due to the ionization effect. Additionally, electrostatic repulsion between the ions caused the polymer chain to diffuse, creating additional space for water molecules within the hydrogel and leading to an increase in the swelling ratio [76].

However, in some conditions or applications, such as underwater sensing, mechanical damage caused by extended swelling can negatively affect the sensing performance of hydrogel-based wearable sensors. Designing nonswellable hydrogels with high mechanical resistance is essential to overcome the disadvantage of excessive water absorption in underwater hydrogel-based wearable sensors [203]. According to Zhao et al. [206], a nonswelling double network hydrogel was developed by incorporating chitosan into a poly(acrylic acid–2–methoxyethyl acrylate) −Fe^3+^ [P(AA–MEA) −Fe] network and used for long-lasting underwater sensing. As a result of its introduction of CS, the hydrogel showed a dense network structure that prevented swelling. As a result of immersion in aqueous solutions (pH = 1, 4, and 7), physiological saline, seawater, and organic solvents, the P(AA–MEA) –CS–Fe hydrogel demonstrated excellent nonswelling characteristics. In another study, a novel kind of swelling-induced enhanced hydrogel was reported with underwater sensing capabilities [221]. Nanocomposite hydrogels produced by combining acrylic acid and 2–acrylamido–2–methylpropane sulfonic acid in the presence of acidic Al(OH)_3_ NPs aqueous solution exhibited relatively weak coordination crosslinking due to the partially prohibited formation of aluminum-carboxylate complexes. As a result of immersion in water, the pH value increased, resulting in strong coordination crosslinking from the aluminum-carboxylate complexation. This resulted in a significant improvement in the mechanical properties of the swollen hydrogel in comparison to the as-prepared sample.

#### 3.3.3. Cycle Life

Cycle life refers to the number of times the biosensor can be used before it reaches the end of its functional lifespan [222]. The cycle life of a hydrogel is an essential parameter in applications where the material is subjected to repeated mechanical loads, such as in tissue engineering scaffolds, drug delivery devices, and wearable biosensors [223]. One of the critical factors that determine the cycle life of hydrogels is their fatigue properties [187]. The fatigue process can occur even when the maximum stress applied to the material is below its ultimate strength [224]. In hydrogels, fatigue can result in a decrease in their cycle life, as well as a decrease in other mechanical properties, such as stiffness and toughness, which can significantly impact the accuracy and reliability of HWEBs. Therefore, fatigue resistance is a critical feature for such devices [225].

Several techniques can be used to measure the cycle life and fatigue properties of hydrogels. One of the most common methods is the uniaxial compression test, where a hydrogel sample is compressed repeatedly at a constant strain or stress level [226]. The number of cycles the hydrogel can withstand before failure is recorded as the cycle life. Another method is the fatigue test, where a hydrogel sample is subjected to cyclic loading with varying stress or strain amplitudes. The material’s mechanical properties, such as stiffness and toughness, are measured before and after the test to evaluate the fatigue damage [187].

The chemical composition of hydrogels, such as the type and concentration of crosslinking agents, can affect their cycle life and fatigue properties. Hydrogels with a higher degree of physical cross-linking typically have a higher cycle life and fatigue resistance [227]. On the other hand, hydrogels with a higher water content are generally more susceptible to fatigue damage than those with lower water content. This is because water molecules can act as plasticizers, reducing the material’s stiffness and making it more prone to failure [226,228].

Temperature can also affect the cycle life and fatigue properties of hydrogels. At higher temperatures, the hydrogel may soften and become more susceptible to fatigue damage. In contrast, at lower temperatures, the hydrogels using pure water as the dispersion medium may become hard and more prone to fracture [229]. The loading conditions, such as the strain or stress level and the loading rate, can significantly affect the cycle life and fatigue properties of hydrogels. It is well known that higher strain or stress levels and loading rates can result in more significant fatigue damage and reduced cycle life.

When it comes to HWEBs, there are also several factors contributing to their fatigue. One of the main factors is the repeated deformation of the hydrogel caused by mechanical stress during daily use. The deformation can lead to changes in the structure and conductivity of the hydrogel, which can affect its sensitivity and durability [230]. Consequently, it is important for HWEBs to endure daily body deformation even under harsh conditions [231].

It is possible to make hydrogels fatigue-resistant by using materials that form multiple hydrogen bonds and chain entanglements that provide more efficient energy dissipation pathways [230]. Polyampholytes are a class of polymers comprising negatively and positively charged monomer subunits with tunable properties controlled by the interaction between their subunits [232]. So, polyampholyte materials can dissipate energy and improve fatigue resistance by relying on weak ionic bonds for reversible breaking and reformation [233]. As a result, to prevent crack propagation when hydrogels are loaded, it is important to introduce efficient energy-dissipating mechanisms into cross-linked networks.

Chen et al. [231] reported a wearable strain sensor using chitosan/poly(acrylic acid-sodium p-styrene sulfonate)/sodium chloride DN hydrogel. To test its resilience and fatigue resistance, the hydrogel underwent five cycles of 200% strain cyclic tensile tests without stopping. Due to the numerous reversible weak molecular interactions presented in the hydrogel, such as hydrogen bonds and electrostatic interactions, the hydrogel demonstrated excellent mechanical properties (elongation at break of 620% and tensile strength of 532.2 kPa), self-recovery, and fatigue resistance even if these bonds were broken during stretching. Sodium chloride was added to the hydrogel to create hydrophobic domains, leading to both the entanglement of the hydrogel macromolecule chains and high elasticity.

Gong et al. [192] developed a composite hydrogel composed of polyvinyl alcohol (PVA), poly(acrylic acid-co-acrylamide) (P(AAc–co–AAm)), and polydopamine-decorated carbon nanotubes. This hydrogel featured a double-physical cross-linked network with two distinct networks. The first network, composed of P(AAc–co–AAm), exhibited relative rigidity, brittleness, and strong cross-linking, enabling significant energy dissipation during the deformation process. The second network, consisting of PVA, provided softness and extensibility to the hydrogel while protecting its overall structure even after the first network experienced fracture. Through ten consecutive cycles of 120% strain tensile tests (Figure 6(bI)), the hydrogel demonstrated an evident hysteresis loop in the first cycle, indicating substantial dissipation of energy (Figure 6(bII)). In the subsequent cycles (second to tenth), the hysteresis loop and dissipated energy remained almost identical, indicating the hydrogel’s ability to recover after loading without undergoing permanent deformation. This conductive hydrogel exhibited an effective energy dissipation mechanism and displayed fatigue resistance due to its internal double-network structure.

#### 3.3.4. Rheological Behavior

Rheology is a preferred technique for characterizing static and dynamic viscoelastic responses of hydrogels [234]. Oscillation mode on a rheometer can measure rheological properties, which describe how materials flow or deform in response to external factors like stress, strain, and temperature over time [235]. The energy stored during deformation or the shear storage modulus (G′), the energy released during deformation or the shear loss modulus (G″), and the loss factor (tan δ = G″/G′) are typically measured for investigating the rheological behavior of hydrogels [236]. If G″ > G′, corresponding to a tan δ > 1, the sample will behave more like a viscous liquid. Conversely, if G′ > G″ (tan δ < 1), the sample will behave more like an elastic solid [237]. Appropriate gelation and cross-linking of polymer networks occur when G′ is much higher than Gʺ, resulting in an elastic storage modulus comparable to soft tissues that can be modulated proportionally by the cross-linker concentration and polymer length [75].

Rheological studies of hydrogel systems are essential to assess their potential for use in HWEBs under external forces, such as shear stress. The role of rheological properties becomes more critical in the case of 3D printable hydrogels. Han et al. [220] conducted a study on the development of a hydrogel comprising gelatin methacrylate (GelMA), sodium alginate (Alg), chondroitin 4-sulfate sodium salt (CS), and sodium carboxymethyl cellulose (NaCMC) (Figure 6(cI)). Their findings highlighted the impact of the GelMA to NaCMC ratio on the formability and mechanical properties of 3D printable hydrogels. Rheological analyses revealed that G′ and G″ remained constant at low strains (<60%) (Figure 6(cII)), indicating a gel-like behavior where G′ exceeded G″. However, as the strain increased within the range of 60–10,000%, G′ exhibited a rapid decrease and approached the intersection with G″, signifying a loss of stability in the gel structure. Specifically, the intersection of G′ curves occurred at a strain of 190% when the GelMA to NaCMC mass ratio was 100:7, resulting in a transition of the hydrogels from a gel to a fluidic state. Notably, an increase in NaCMC content led to a decrease in the strain value at the junction of G′ and G″. Consequently, composite hydrogels with a higher NaCMC content displayed increased instability and were unsuitable for extrusion printing.

Another study demonstrated an improvement in the rheological properties of the PE-DOT:PSS solution by incorporating a hydrophilic 2D material, Ti_3_C_2_ MXene, into the ink formulation. This modification resulted in the formulation of a fully aqueous ink suitable for extrusion printing while also enhancing the conductivity of the 3D-printed hydrogels. The MXene nanosheets possessed a chemically rich surface and rigidity, facilitating interactions with PEDOT:PSS. These interactions promoted the redistribution of PEDOT:PSS within the ink, thereby enhancing the ink properties for printing, as illustrated in Figure 6d [66].

### 3.4. Electrochemical Techniques

In HWEBs, hydrogels are an innovative and promising material with significant potential for further investigation. There are two groups of hydrogel responsiveness: the first being carriers that respond passively to pH dependence and electrical dependence. The second group can respond to the presence of a compound, such as a biomarker or bioanalyte, in the surrounding environment when they become functionalized [238]. Electrochemical sensing mechanisms, including Potentiometric, Amperometric, Conductimetric, and Impedimetric methods, are commonly used in HWEBs [239]. There are several differences between these methods, including electrode configurations, applied and measured signals, and mass transport regimes. Signals can be constant or time-varying, and thus, electrochemical responses can be transient, steady-state, or both [240]. Measurements of these methods are indicative of the local activity within nanometers of the electrode surface [239]. There are several factors that must be taken into consideration when choosing the appropriate electrochemical sensing method for detection, such as the type of analyte to detect, the sensitivity and selectivity requirements, the sample matrix, as well as the instrumentation available [241].

#### 3.4.1. Current Response

Voltammetry and amperometry are two rapid and real-time methods used to detect biomarkers, biomolecules, drugs, etc., in HWEBs [242,243]. Amperometric biosensors measure currents generated by electrochemical reactions related to the oxidation or reduction of electroactive species [244]. The resulting current has a direct correlation with the bulk concentration of electroactive species or their production rate within the adjacent biocatalytic layer [244]. Voltammetry methods involve the measurement of a current in response to an electrical potential [240]. Linear sweep voltammetry (LSV) involves measuring the current generated by an applied electrical potential that is continuously varied across a range of potentials. In cyclic voltammetry (CV), the electrical potential is swept in both forward and reverse directions in partial cycles, full cycles, or series of cycles [240]. Pulse voltammetry applies the electrical potential in pulses, which increases signal sensitivity compared to conventional voltammetric methods, such as LSV and CV [245]. It is possible to separate the capacitive current from the faradic current by pulse voltammetry since the capacitive current is decreasing to zero value. Therefore, faradic currents related to redox spices can be detected more accurately [246]. Staircase voltammetry involves applying a series of stair-step voltage pulses, with the current measured after each step change. Square wave voltammetry (SWV) is a specific type of staircase voltammetry where a symmetric square-wave pulse is added to the staircase potential waveform. In SWV, the current response to the applied potential is pulsed once in the forward direction and once in the reverse direction. This technique helps discriminate the charging current and reduces it compared to rapid scanning CV [247]. Differential pulse voltammetry (DPV) is another voltammetric technique where the electrical potential is scanned using a series of fixed-amplitude pulses superimposed on a changing base potential [240]. DPV is similar to SWV but offers increased discrimination of Faradaic currents due to potential perturbations created by small pulses [247]. Therefore, it can be concluded that the pulse techniques take advantage of minimum non-faradic currents and enhance the sensitivity of electrochemical biosensors.

**Figure 7:**
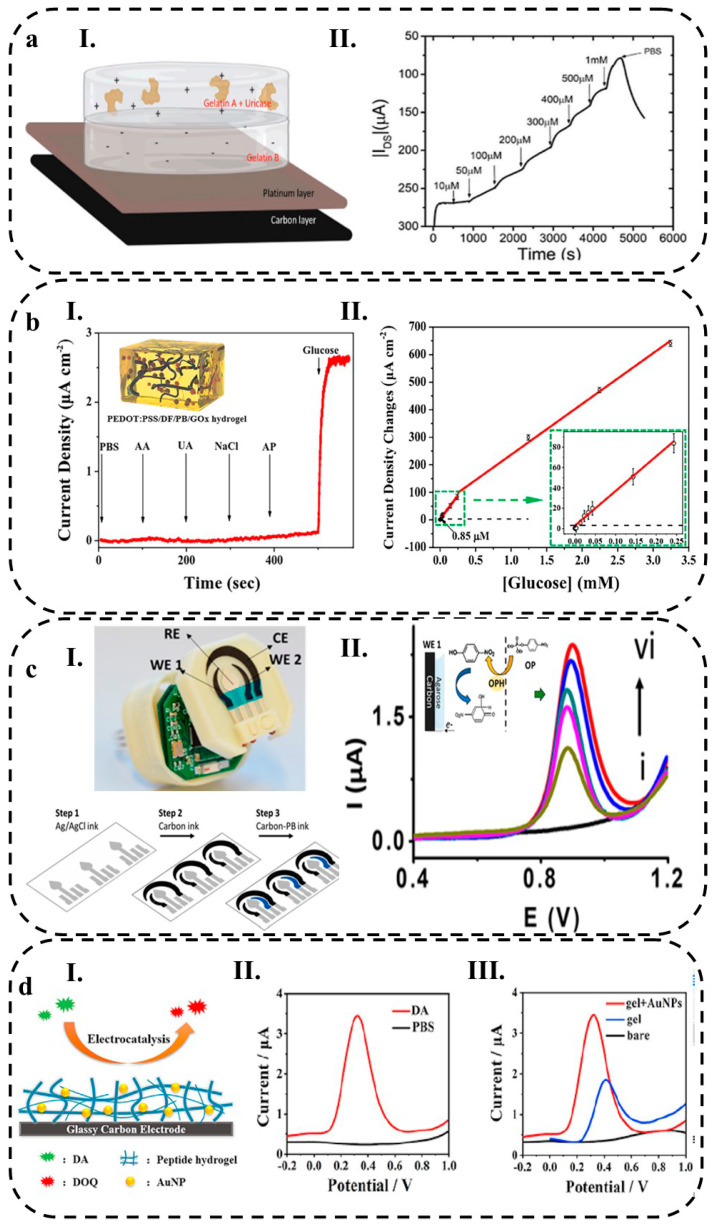
(**aI**) Two layers of gelatin hydrogels integrated on the gate electrode surface, comprising a platinum layer and a carbon layer, (**aII**) The amperometric response to changes in UA concentrations in a PBS solution. Reprinted with permission from Wiley from ref. [248], (**bI**) The amperometric response of the PEDOT:PSS/DF/PB/GOx sensor during a glucose selectivity test, (**bII**) The calibration curve for the PEDOT:PSS/DF/PB/GOx sensor used for glucose detection. Reprinted with permission from Elsevier from ref. [51], (**cI**) The hydrogel-based ring case, including the carbon working electrode (WE 1), Ag/AgCl reference electrode (RE), carbon/PB working electrode (WE 2), and carbon counter electrode (CE), (**cII**) The SWV response for different concentrations of MPOx from 0 to 1.25 mM in 0.1 M PBS, with 0.25 mM increments (black, brown, purple, green, blue and red lines show 0, 0.25, 0.5,0.75, 1 and 1.25 mM of MPOx, respectively). The inset shows the corresponding calibration plot. Reprinted with permission from ACS from ref. [249], (**dI**) A schematic representation of the peptide/AuNPs hydrogel electrode used for the catalysis of DA, (**dII**) The DPV curves of the peptide/AuNPs hydrogel electrode in the presence (red) and absence (black) of 1.0 mM DA in 10 mM PBS, (**dIII**) The DPV curves of 1.0 mM DA recorded at the bare GCE (black), peptide/GCE hydrogel (blue), and AuNPs/peptide/GCE hydrogel (red). Reprinted with permission from Elsevier from ref. [250].

Galliani et al. [248] developed a printed organic electrochemical transistor for the detection of UA. The device incorporated two layers of hydrogels on the surface of the gate electrode (Figure 7(aI)). These hydrogel layers consisted of a polycation and a polyanion network, creating a charge-selective barrier that prevents the passage of charged molecules to the gate electrode. This barrier effectively suppresses parasitic Faradaic reactions resulting from the oxidation of electroactive molecules in the measuring medium. Consequently, only H_2_O_2_ is allowed to reach the gate, enabling selective detection of UA. The sensor utilized the amperometric method for UA detection, as depicted in Figure 7(aII), which shows the real-time response to increasing UA concentrations in PBS (phosphate-buffered saline). The sensor demonstrated high sensitivity within the range relevant to pathological levels of UA in wounds (<200 μM). It achieved an LOD of 4.5 μM (Table 2) when tested in an artificial wound exudate.

In another research by Xu et al. [51] amperometry technique was employed for electrochemical glucose detection. They utilized a PEDOT:PSS hydrogel as a conductive matrix to incorporate PB NPs and GOx onto glass carbon electrodes. To improve the electron transfer rate and ensure uniform distribution of PB NPs within the hydrogel, DMSO and Zonyl FS–300 were employed to extend the polymer chains of PEDOT:PSS. The hydrogel-loaded electrodes were applied as skin patches for in vivo monitoring of glucose levels in human subjects. The current responses of other interferents like AA and UA were found to be insignificant compared to the response of glucose, as illustrated in Figure 7(bI). The calibration curve (Figure 7(bII)) demonstrated that PEDOT:PSS/DF/PB/GOx exhibited a competitive sensitivity of 340.1 μA mM^−1^ cm^−2^ within a linear range of 1 to 243 μM (Table 2).

A novel wireless and wearable platform in the form of a ring has been developed for the rapid monitoring of explosive and nerve agent threats in both vapor and liquid phases. This platform integrates printed electrochemical sensors and a miniaturized electronic interface for wireless data transmission. Dual-working electrodes were printed using carbon and carbon/PB inks. The utilization of carbon/PB inks enabled the detection of explosives and nerve agents, making them suitable for surveillance applications. To enhance analyte diffusion to the electrode, the sensor surface was modified with a semisolid agarose hydrogel coating (Figure 7(cI)). For detection purposes, the SWV technique was employed. Figure 7(cII) displays the SWV results obtained for five increments of 0.25 mM concentration of methyl paraoxon (MPOx) in PBS ranging from 0.25 to 1.25 mM. The anodic peaks observed correspond to the oxidation of the nitrophenol product generated from the enzymatic reaction between MPOx and organophosphorus hydrolase (OPH). Notably, the peak current exhibits a linear relationship with the MPOx concentration, and the LOD achieved in this study was 200 μM (Table 2) [249].

As an example, Wang et al. [250] conducted a recent study where they developed a peptide hydrogel with excellent biocompatibility. To inhibit bacterial growth, the antibiotic ciprofloxacin (CIP) was selected as the doping drug within the hydrogel. Additionally, the incorporation of AuNPs was utilized to enhance the specific surface area and conductivity of the hydrogel (Figure 7(dI)). The peptide/AuNPs hydrogel was evaluated for its catalytic ability in converting the neurotransmitter dopamine (DA) into dopamine quinone. Figure 7(dII) demonstrates that no peak current was observed in phosphate-buffered saline alone, but when DA was added, a peak current appeared in the differential pulse voltammetry (DPV) test. The LOD achieved was 21 nM, with a linear range of 0.1 to 10 μM (Table 2). Furthermore, when a GCE was modified with the peptide/AuNPs hydrogel, it exhibited significantly improved electrochemical catalytic performance compared to the bare GCE and GCE modified with peptide hydrogels alone. This enhancement is illustrated in Figure 7(dIII), where an increase in oxidation peak current is observed. The improved electrochemical performance can be attributed to the large specific surface area and high conductivity of the peptide hydrogel. Additionally, the negatively charged peptide hydrogel attracted the positively charged dopamine, further enhancing the electrochemical response.

#### 3.4.2. Potential Response

Potentiometry is a widely used electrochemical technique for measuring the potential difference between typically two electrodes in a solution. Potentiometric biosensors consist of sensing electrodes and a reference electrode, allowing for direct detection of targets via the measurement of the potential signal generated by the change in surface charge at the sensing electrode upon target identification [241]. Usually, an ion-selective electrode (ISE) is used, which has an ion-selective membrane that selectively responds to specific ions, such as K^+^, Ca^2+^, H^+^ (pH sensor), etc. The ISE is polarized at a fixed potential, and the reference electrode (RE) is used to measure the potential difference between the sensing electrodes. The potential of RE is constant and independent of analyte concentrations, while ISE depends on it, leading to analyte concentrations being determined based on the potential differences between the ISE and RE [242]. Open circuit potentiometry (OCP) which doesn’t use an external power source, is based on the selective binding of target molecules to the surface of the sensing electrode. This leads to a change in the surface potential and, thus, a measurable signal. One of the advantages of this method is that potentiometry has a high impedance, which minimizes interference and sensor fouling. Potentiometry is less sensitive to electrode size and has a slow fouling rate. Therefore, potentiometric sensors are ideal for continuous sensing applications [266].

**Figure 8:**
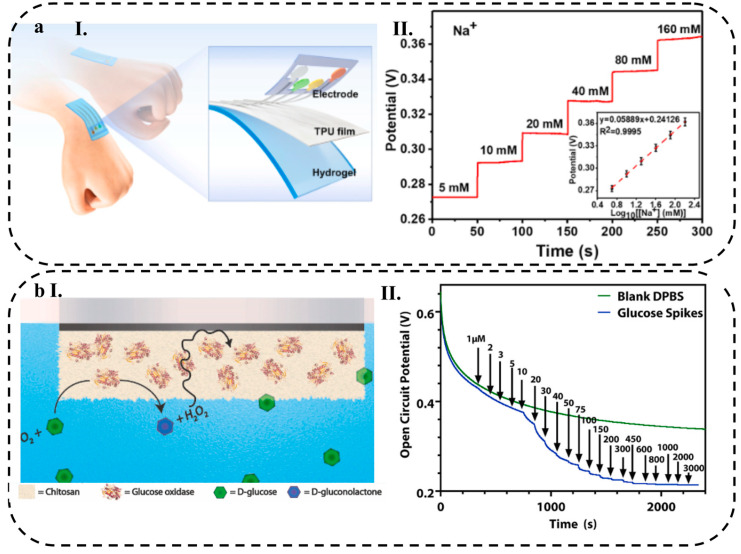
(**aI**) Schematic illustration of an HWEB using hydrogel-elastomer materials, (**aII**) OCP curve for HWEB at various Na^+^ concentrations shown insets is the calibration plot for the sensor. Reprinted with permission from Elsevier from ref. [265], (**bI**) the surface reaction of the GOx-chitosan on platinum electrode, and (**bII**) GOx-chitosan on platinum macroelectrode OCP in a blank solution of DPBS (green), and in a solution of DPBS containing glucose with various concentrations (blue). Reprinted with permission from Elsevier from ref. [266].

Wang and colleagues [265] proposed a wearable patch for monitoring biofluids, which is designed as an attachable skin-like structure. This patch utilizes a hydrogel-elastomer system and incorporates a thin thermoplastic polyurethane (TPU) film to connect stretchable hydrogels with conductive ink. This configuration ensures both good electrical conductivity and mechanical flexibility (Figure 8(aI)). The patch includes a device for OCP detection, allowing for the measurement of pH, Na^+^, and K^+^ levels. The pH measurement demonstrated a sensitivity of 58.14 mV pH^−1^, while Na^+^ and K^+^ measurements exhibited sensitivities of 58.89 and 59.11 mV decade^−1^, respectively. The LOD for Na^+^ and K^+^ was determined to be 7.94 and 5.37 μM, respectively (Table 2). In Figure 8(aII), the OCP of a Na^+^ sensor is depicted, demonstrating its performance in detecting physiologically relevant concentrations of Na^+^ (ranging from 5 to 160 mM). The sensor exhibits good linearity and sensitivity in Na^+^ detection.

In a recent study, an OCP HWEB was developed by electrodepositing chitosan polymer films on platinum electrode surfaces in the presence of GOx. Chitosan is used due to its unique properties, such as inexpensiveness, biocompatibility, non-toxicity, and ease of use. As shown in Figure 7(bI), with the enzyme presence, chitosan encapsulated GOx near the electrode surface. When the GOx meets its substrate, oxygen is consumed, and hydrogen peroxide is generated. The OCP technique is then used to detect changes due to oxygen consumption and the generation of hydrogen peroxide (Figure 8(bII)). The linear range of 1–10, 10–100, and 100–3000 μM, with sensitivity 0.5, 0.012 and 0.19 mV/μM, were obtained, respectively, for each linear range (Table 2). A GOx-chitosan electrode biosensor was calibrated repeatedly over a period of 28 days to determine its stability. A minimal change in the sensor’s performance was observed, indicating that it can be used for at least a month [266].

#### 3.4.3. Conductometry

Conductometry is an electrochemical technique measuring the conductivity of a solution. The conductometric biosensor utilizes a low-amplitude alternating electrical potential to measure the ability of an analyte to conduct current between two electrodes [267]. The target analyte changes the electrical conductivity of the solution resulting from the production or consumption of charged species. This change in conductivity is detected and measured by the biosensor [240]. Up to date, few conductometric wearable sensors have been reported due to the use of high-capacity power sources and large electronics [242]. A number of studies, however, use the conductivity change and resistance change principles. In these examples, the change in resistance is due to the chemical reaction between the target analyte and the sensing material, which alters its electrical properties.

**Figure 9:**
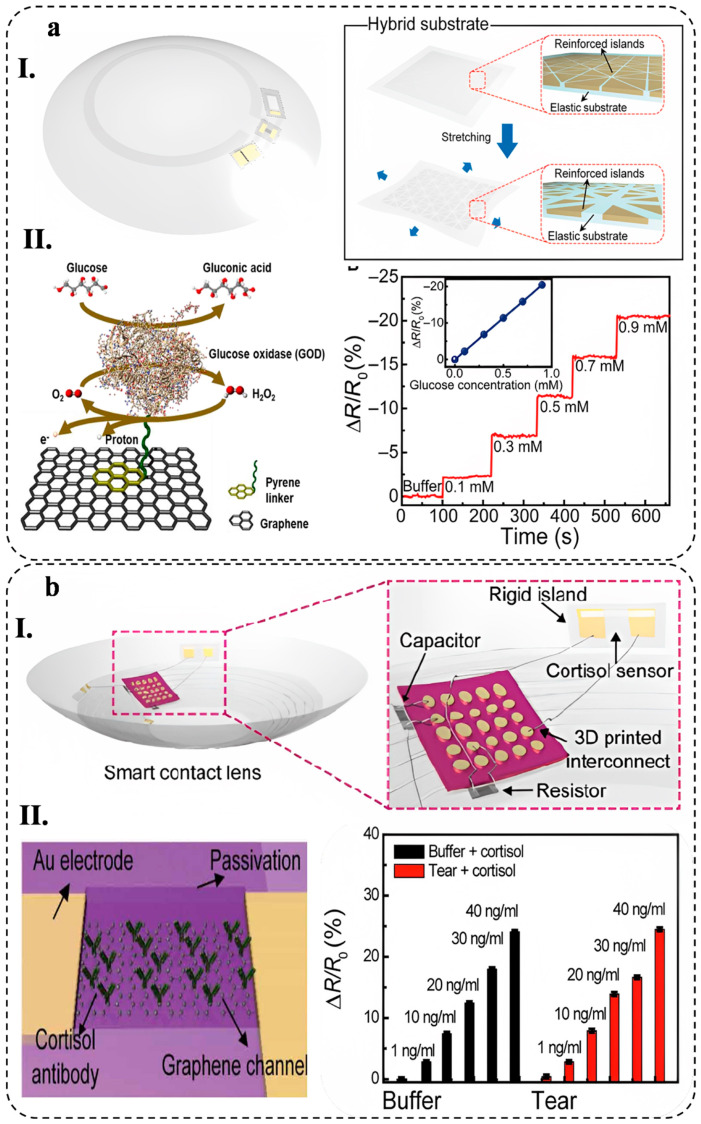
(**aI**) Schematic illustration of contact lens consisting of a PI and PDMS hybrid substrate, (**aII**) The relative resistance change response of the contact lens sensors with different glucose concentrations, inset is calibration curves of the glucose sensor. Reprinted with permission from Science from ref. [261], (**bI**) A schematic illustration of a packaged smart contact lens consisting of NFC chip, capacitor, resistor, antenna and cortisol sensor, and (**bII**) Change in relative resistance with increasing concentrations of cortisol in the buffer (black) and aqueous solution of artificial tears (red). Reprinted with permission from Science from ref. [262].

Park et al. [261] introduced a smart contact lens fabricated from a hybrid substrate that combines a stretchable, transparent antenna with a glucose sensor. Polyimide (PI) and polydimethylsiloxane (PDMS) layers were used as substrates. A silicone elastomeric layer (LENS) was applied to the substrate. The glucose sensor was made of platinum (Pt) nanoparticles embedded in a conductive polymer matrix (Figure 9(aI)). GOx was immobilized on the graphene surface with pyrene linkers through π-π stacking interactions. GOx oxidizes glucose resulting in product passing through the graphene channel, producing H_2_O_2_ as a byproduct. As H_2_O_2_ decomposes, oxygen, protons (H^+^ ions), and electrons are generated. Protons are responsible for the positive charge transfer effect of graphene channels. Thus, the sensor’s relative change in resistance (∆R/R0) can be detected as a function of glucose concentration. The resulting contact lens measured the glucose concentration in tears using conductometry (Figure 9(aII)). The sensor exhibited a LOD of 12.57 μM and a sensitivity of 22.72% mM^−1^ (Table 2).

In another investigation, a soft contact lens was developed to integrate a cortisol sensor along with transparent antennas and wireless communication circuits. This design allows for the remote operation of the sensor via a smartphone without obstructing the wearer’s vision. The flexible segments of the smart contact lens are constructed using silicone elastomer, a conventional material for soft lenses. The contact lens device comprises an NFC chip, capacitor, resistor, antenna, and cortisol sensor, which incorporates a graphene field-effect transistor (Figure 9(bI)). The smart contact lens incorporates an immunosensor specifically designed for cortisol detection. In this approach, graphene field-effect transistors are chemically bonded with C-Mab to create the immunosensor. The conductivity of the graphene channel within the field-effect transistor varies with changes in cortisol concentration. As the cortisol concentration increases, the carrier concentration in the graphene channel decreases, leading to a reduction in drain current (Figure 9(bII)). The method achieved an LOD of 10 pg mL^−1^ (Table 2) [262].

#### 3.4.4. Impedance Response

Impedimetric methods, also known as frequency response methods, rely on analyzing the response of a system to periodic applied current or potential waveforms either at a fixed frequency or over a range of frequencies [240]. EIS is a highly sensitive technique for detecting molecular interactions near electrode surfaces. Due to its ability to capitalize on the intrinsic properties of molecules, this approach is an ideal tool for detecting specific molecules, such as hormones, proteins, nucleic acids, and metabolites. EIS provides researchers with the ability to study reaction mechanisms and estimates analyte concentrations in real-time [268]. The EIS process involves the acquisition of a Nyquist plot and a Bode plot. The Nyquist plot displays the real impedance and imaginary impedance (*x*- and *y*-axis, respectively) at various frequencies. On the other hand, the Bode plot represents the logarithmic frequency plotted against the logarithmic complex impedance and phase angle. Analyzing these plots provides valuable insights into the behavior of electrochemical systems, including their resistance and capacitance [269]. One notable advantage of the EIS method is its compatibility with integrated circuits, enabling the development of portable and cost-effective wearable platforms. These sensors can be seamlessly integrated into wearable technology, facilitating non-invasive detection. This integration offers advantages such as cost-efficiency and user-friendliness [241,268].

**Figure 10:**
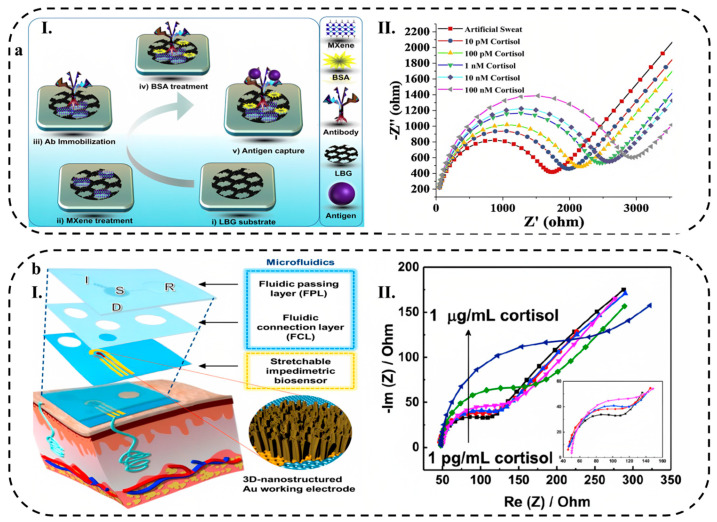
(**aI**) Schematic for Ti_3_C_2_T_x_ MXene-loaded/LBG-based cortisol biomarker detection, (**aII**) Nyquist plot for cortisol biomarker detection Ti_3_C_2_T_x_ MXene-loaded/LBG-based using EIS method. Reprinted with permission from Elsevier from ref. [264], (**bI**) lab-on-a-patch platform exploded view, and (**bII**) The Nyquist plots for the stretchable impedimetric biosensor (black, red, blue, purple, green and dark blue curves show cortisol concentrations of 0.001, 0.01, 0.1, 1, 10 and 1000 ng mL^−1^, respectively). Reprinted with permission from Elsevier from ref. [263].

In a study by Nah et al. [264], a wearable electrochemical impedimetric immunosensor was made of laser-burned graphene (LBG) flakes. This sensor is constructed on PDMS, a flexible and elastic substrate. A PDMS substrate is coated with laser-burned graphene (LBG), which is transferred by removing a polyamide (PI) film. As a result, sweat is allowed to flow through a porous structure. The LBG electrode is loaded with highly conductive Ti_3_C_2_T_x_ MXene to improve its electrochemical performance. A microfluidic system is fabricated using a 3D-printed mold and PDMS. The sweat is collected from the skin and moved through a channel to a sensor chamber. Cortisol binding to an antibody on the sensor surface causes changes in electrical impedance and provides real-time monitoring of cortisol levels in sweat (Figure 10(aI)). According to the Nyquist plots in Figure 10(aII), the charge transfer resistance (R_ct_) values gradually increased with increasing cortisol concentrations. As more cortisol molecules bind to the antibody on the surface of the sensor, electron transport across the interface between the electrode and electrolyte solution becomes more difficult, resulting in an increase in R_ct_. The Ti_3_C_2_T_x_ MXene/LBG/PDMS-based patch cortisol immunosensor demonstrated linearity of 0.01–100 nM (Table 2). This impedimetric immunosensor displayed a very low LOD of 3.88 pM.

Lee and colleagues [263] developed a wearable biosensor that can detect cortisol in body fluids in a noninvasive and quantitative way. This platform consists of a stretchable impedimetric biosensor with a 3D-nanostructured Au working electrode. Using the biosensor integrated with microfluidics, sweat can be collected autonomously, and the reagent delivered using one-touch finger operation for the measurement of impedimetric signals (Figure 10(bI)). The Nyquist plots in Figure 10(bII) show that different R_ct_ values were obtained with various cortisol concentrations. 1 pg mL^−1^ and 0.25 Ω ng^−1^ mL^−1^ are representative of the LOD and sensitivity for cortisol detection, respectively.

#### 3.4.5. Benefits and Drawbacks of Electrochemical Techniques

Although each electrochemical technique has its own advantages and disadvantages, they can all be used to create wearable biosensors. The amperometry biosensor can identify the substance with the detection of a current produced by an oxidation or reduction reaction of the analyte. This method is excellent for identifying analytes at low concentrations because of its great sensitivity [241]. Nevertheless, prone to disruption from other electroactive species in the sample [270]. A voltammetry biosensor detects analytes by applying a potential difference across an electrode and measuring the resulting current. Due to its capacity to discriminate between various electroactive species, it can identify particular analytes in complex samples [271]. However, it may not be commercially viable due to its large size and high-cost [241]. Another drawback of amperometry and voltammetry is the dependence of the signal (current, *i*) on the electrode size, which may change over time due to fouling [266].

A potentiometry biosensor measures change in electrode potential to identify analytes. Despite its simplicity and cost-effectiveness, this method is not as sensitive as amperometry methods and is susceptible to interference from other electroactive species. The potentiometry method is often used to detect ions, such as pH and other electrolytes, but may not be effective for detecting molecules that require a higher level of sensitivity, such as small molecules or proteins [241].

Conductometry biosensors monitor the electrical conductivity of solutions or hydrogels to identify analytes. Furthermore, conductometry does not need a reference electrode and can be used to detect both electro-active and inactive analytes [240]. Temperature, pH, and other electrolytes in the sample can all impact conductometry, so it is less sensitive than other techniques [272]. Due to the use of high-capacity power sources and large electronics, research on wearable conductometry is rare [242].

EIS is an excellent method for identifying specific compounds, including hormones, proteins, nucleic acids, and metabolites. This is because it is highly sensitive to molecular interactions near an electrode surface. With EIS, biomolecular interactions can also be detected in real-time using the impedance interface as a function of time [240]. It is, however, in practical applications limited by the non-specific binding of non-target compounds, which results in a low level of sensitivity and selectivity [241].

Electrochemical techniques for wearable biosensors are largely determined by the application and requirements of the device. Therefore, a number of factors must be considered, including sensitivity, selectivity, power requirements, and cost.

## 4. Applications of HWEBs

HWEBs are gaining popularity due to their numerous potential applications in various fields, including medical, environmental, and industrial applications. They have the potential to revolutionize the way biological parameters are monitored. In this section, we discuss the current state-of-the-art applications of HWEBs.

### 4.1. HWEB Platforms

There are a variety of platforms that have been developed for HWEBs, such as wearable patches [71], epidermal tattoos [273], microfluidic-based platforms [274], microneedle–based platforms [275], soft contact lens [276], and paper-based HWEBs [277]. Each of these platforms has unique advantages and disadvantages, and the choice of platform depends on the specific application and requirements of the device.

#### 4.1.1. Wearable Patches

Wearable patches are flexible and conformable devices that can provide a stable and non-invasive interface for monitoring various biological signals, which can be applied directly to the skin [71,278]. Hydrogel-based wearable patches have been widely studied for their potential use in the electrochemical biosensors [239]. These devices are ideal for the continuous monitoring of biological parameters [71].

**Figure 11:**
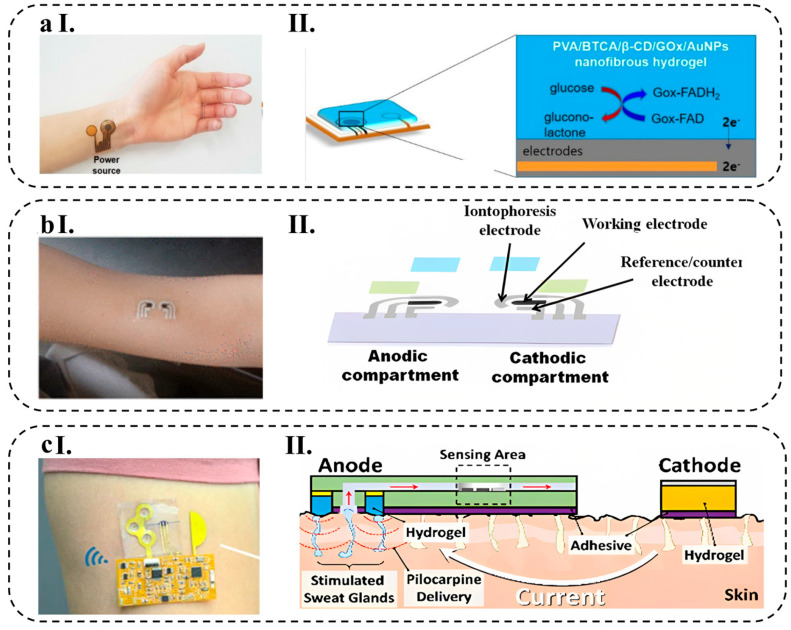
(**aI**) Schematic illustration of the hydrogel-based patch for glucose monitoring, (**aII**) Working mechanism diagram using PVA/BTCA/β-CD/GOx/AuNPs nanofibrous hydrogel. Reprinted with permission from Nature from ref. [71], (**bI**) GOx-functionalized hydrogel tattoo, (**bII**) Working mechanism diagram. Reprinted with permission from ACS from ref. [273], (**cI**) using hydrogel in epidermal electrochemical microfluidic biosensor for monitoring of various analytes in sweat, and (**cII**) Working mechanism diagram. Reprinted with permission from Springer from Ref. [274].

Transparent microfiber and nanofiber hydrogel patches are discussed by Jin et al. [71] for use in monitoring interstitial glucose levels continuously (Figure 11(aI)). The team created a new hydrogel using poly(vinyl alcohol)/β-cyclodextrin polymer that is transparent and flexible, making it well-suited for biosensor applications. These hydrogels exhibit a range of desirable properties, including excellent absorptivity, good mechanical properties, and high enzyme activity (Figure 11(aII)). Hydrogels are a suitable choice for biosensors because they possess excellent properties, such as high permeability and rapid electron transfer. This biosensor was found to have excellent sensing performance, with a wide linear range, high sensitivity (47.2 μA mM^−1^), low sensing limit (10 μM), and rapid response time (<15 s). In future clinical applications, the biosensor could be used to accurately measure glucose concentrations in human serum.

#### 4.1.2. Epidermal Tattoos

Epidermal tattoos are being developed to meet the growing demand for non-invasive, continuous monitoring of biological parameters in real time. They are thin, flexible, and biocompatible devices designed to be worn directly on the skin like a temporary tattoo, which provides a stable and robust platform that is easy to use for the detection of biological signals which causes no pain or discomfort [273,279]. One of the main advantages of the tattoo platform is its non-invasive design. The tattoo platform can be worn without causing any discomfort or pain. This makes it ideal for use in long-term monitoring applications, where the user needs to wear the device for extended periods of time [280]. Another key benefit of using hydrogel-based tattoos as a platform for wearable biosensors is their electrochemical nature, which allows for the direct measurement of target analytes [19]. The tattoo platform is connected to a wearable device that contains the electronic components required to process the data from the biological materials [273,281].

Bandokar et al. [273] reported the development of a hydrogel-based tattoo glucose sensor for non-invasive electrochemical monitoring of glucose levels (Figure 11(bI)). The sensor is designed to be worn as a temporary tattoo and is the first of its kind to combine reverse iontophoretic extraction of interstitial glucose with an enzyme-based amperometric biosensor. The device can be easily worn on the skin and has the potential to provide continuous glucose monitoring without the need for finger-stick blood tests with a sensitivity of 23 nA μM^−1^ and a low limit of detection (3 μM) (Figure 11(bII)). In vitro, studies were conducted to evaluate the sensor’s performance, and the results showed that the tattoo sensor has a linear response to physiologically relevant glucose levels with minimal interference from other electroactive species commonly found in interstitial fluid.

#### 4.1.3. Microfluidic-Based Platforms

Microfluidic-based platforms combine the properties of hydrogels and microfluidics to create non-invasive wearable devices that can monitor various biological signals in real-time with high precision [282]. The use of hydrogel material in these biosensors also offers the potential for improved comfort and skin adherence [274]. These features make microfluidic platforms ideal for the development of wearable biosensors that can be used to monitor a variety of biological signals [274,283].

Bolat and colleagues [274] have developed a hydrogel-based wearable epidermal microfluidic device (Figure 11(cI)) that facilitates the stimulation, collection, and analysis of sweat through a transdermal pilocarpine delivery system. This device is specifically designed for soft skin-mounted applications and offers non-invasive interfaces for on-demand sweat sampling and analysis. The microfluidic channel within the device enables real-time electrochemical monitoring of sweat glucose, and its layout was optimized using fluid dynamics. To enhance usability and functionality, the device was integrated with a wireless data transmission system. This integration enables the device to perform in real-time and allows for the monitoring of biomarkers in stimulated sweat samples, making it suitable for a range of healthcare and wellness applications (Figure 11(cII)).

#### 4.1.4. Microneedle-Based Platforms

Microneedle-based platforms are a promising technology for the development of HWEBs, as they are stable, biocompatible, small, and minimally invasive. Furthermore, they can penetrate through the skin and monitor biological analytes painlessly and continuously [284,285]. The integration of microneedles with HWEBs provides a solution for continuous and non-invasive monitoring of a range of analytes, including glucose [284], lactate [286], pH [287], and body electrolytes [288]. They also offer a range of benefits, such as improving skin permeability [289], increasing wear comfort [290], and reducing the risk of irritation and discomfort for the patient [291].

The microneedles serve as a point of contact with the interstitial fluid, which can be used as a sample source for electrochemical analysis [292]. Such microstructures are small enough to be painless yet large enough to allow for efficient fluid exchange, making them ideal for long-term monitoring [293].

**Figure 12:**
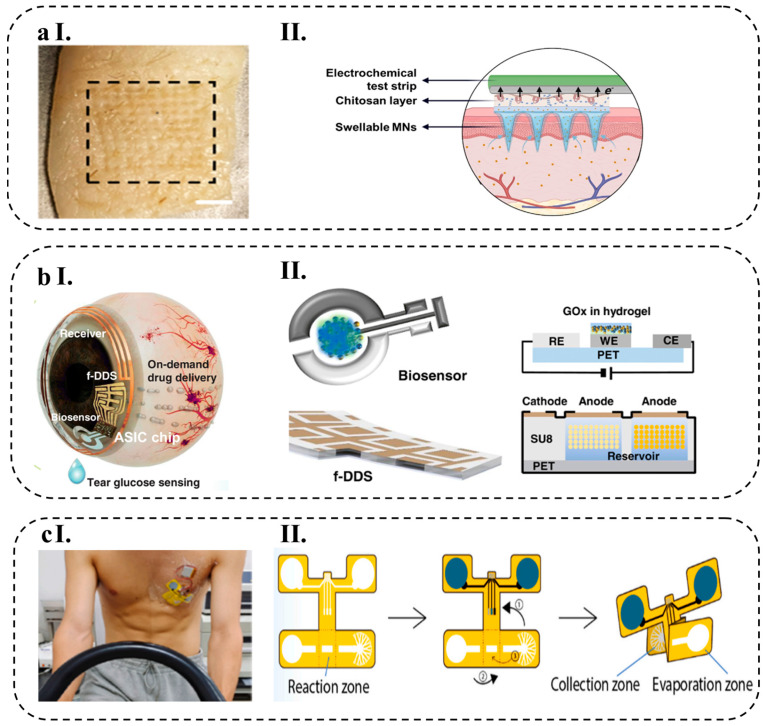
(**aI**) swellable microneedle patch made of hydrogel, (**aII**) Working mechanism diagram. Reprinted with permission from Wiley from ref. [284], (**bI**) a smart contact lens for tear glucose level measurement, (**bII**) Working mechanism diagram. Reprinted with permission from Science from ref. [276], (**cI**) hydrogel-paper patch that simultaneously detects electrophysiology and biochemical changes during exercise, and (**cII**) Working mechanism diagram, by using origami techniques (where steps (1), (2), and (3) represent the folding process), a flat piece was transformed into a 3D configuration of wearable biosensor including a region for collecting sweat, an area where glucose sensing takes place, and a zone for evaporation. Reprinted with permission from Elsevier from ref. [277].

Zheng and colleagues [284] developed an innovative microneedle-based device that enables the monitoring of biomarkers, such as glucose and alcohol, in the interstitial fluid of the skin (Figure 12(aI)). This device consists of expandable microneedles and electrochemical test strips, which facilitate the accurate detection of glucose using an agarose hydrogel. The microneedles are connected to the test strips through a chitosan layer and are designed to penetrate the skin, allowing the extraction of ISF that subsequently flows to the test strip for analysis (Figure 12(aII)). Through in vitro experiments, the device demonstrated precise detection of glucose concentrations ranging from 0 to 12 mM and alcohol concentrations ranging from 0 to 20 mM. In addition, in vivo testing revealed the device’s capability for minimally invasive sampling of ISF and analysis of glucose levels in mice. The authors propose that this microneedle-based device offers a cost-effective and convenient approach for researchers to extract skin ISF for biomarker analysis.

#### 4.1.5. Soft Contact Lens

Electrochemical Soft contact lenses are a new type of wearable biosensors that utilize tears as a biological fluid for detecting various analytes [294]. These biosensors are based on the principle of electrochemistry and can measure the electrical signals generated by the interactions between the biomolecule and bioreceptor [294,295].

Soft contact lens biosensors have several advantages over traditional wearable biosensors. First, they are non-invasive and painless, making them suitable for monitoring of biological parameters [1,276]. Second, they are biocompatible, which means they do not cause any adverse reactions when in contact with biological fluids [296]. Third, they have a high sensitivity and specificity, which allows for accurate and reliable measurement of the target analyte [1]. Despite their advantages, there are also some challenges associated with soft contact lens biosensors. One of the major challenges is the limited lifetime of the biosensor, which is a result of the gradual degradation of the hydrogel matrix and the sensing layer over time [296].

A smart contact lens can act as an effective and convenient interface between the human body and an electronic device for wearable healthcare applications. Keum et al. [276] developed a smart contact lens that can be used for continuous glucose monitoring and treating diabetic retinopathy (Figure 12(bI)). The device is made of a biocompatible polymer and contains thin, flexible electrical circuits, a microcontroller chip, and wireless communication for real-time electrochemical biosensing, on-demand controlled drug delivery, wireless power management, and data transmission (Figure 12(bII)). Using diabetic rabbit models, the researchers demonstrated that tear glucose levels measured by the smart contact lens were consistent with those obtained by conventional invasive blood glucose tests. They also showed that drugs could be released from reservoirs in response to electrochemical signals to treat diabetic retinopathy. Tear glucose in the range of 0 to 49.9 mg dL^−1^ can be measured accurately by employing this soft contact lens. This study successfully demonstrated the potential of smart contact lenses for noninvasive and continuous diabetic diagnosis and treatment.

#### 4.1.6. Paper-Based and Textile-Based Platforms

Paper-based platforms and conductive papers have been identified as attractive options for wearable sensing applications due to their low cost, scalability, ease of disposal, and capillary transport capabilities. However, the paper microfluidic channel on these devices must be replaced frequently since they do not allow for long-term biomarker measurements [297,298]. Textiles are another promising platform for HWEBs, offering flexibility, versatility, breathability, stability, non-invasiveness, and comfort during wear [282,299,300]. A paper-based hydrogel electrochemical wearable biosensor is made by designing and fabricating the biosensor onto a paper-based substrate, preparing a hydrogel, and immobilizing a biomolecule onto it to detect the target analyte. Textile-based biosensors utilize hydrogel, which is soft, pliable, and biocompatible. This hydrogel can be functionalized with specific enzymes or other biomolecules to selectively detect specific analytes of interest [301].

Li et al. [277] developed an integrated and flexible hydrogel-based electrochemical paper patch that simultaneously detects electrophysiology and biochemical changes in sweat during exercise (Figure 12(cI)). The paper patch was self-assembled by a porous PEDOT:PSS hydrogel on a paper fiber, enabling it to serve as an electrocardiogram electrode with low impedance and a glucose sensor with ultra-high sensitivity (1018.2 μA mM^−1^ cm^−2^) and low LOD of 10.3 μM (Figure 12(cII)). Additionally, it provides excellent conductivity and hydrophilic properties, which are responsible for electron transmission and substance diffusion, respectively.

### 4.2. Biosensing Applications

IUPAC defines a biosensor as a device that detects chemical compounds, usually with electrical signals resulting from specific biochemical reactions mediated by enzymes, immune systems, tissues, organelles, or whole cell [302]. Biological components consist of enzymes, antibodies, nucleic acids (DNA/RNA), whole cells, etc., while physical components include transducers converting biological signals into electrical signals to be detected by electrical devices [303]. The biological recognition element of EBs specifically binds to the analyte of interest, and the transducer (e.g., electrodes) transfers the generated electrical signal to the electrical reader device, determining the concentration of the analyte [304]. These wearable devices incorporate electrodes made from conductive materials that are integrated into the hydrogel matrix. The electrodes are used to detect the concentration of various biochemicals, including glucose, lactate, and urea, among others, using electrochemical methods [257,305]. The integration of the electrodes into the hydrogel matrix allows for stable and long-term monitoring of biochemicals, as the hydrogel provides a protective layer that helps maintain the stability of the electrodes and prevents their degradation over time [239].

#### 4.2.1. Catalytic HWEBs

Catalytic HWEBs consist of a hydrogel matrix and a biological enzyme, which acts as a catalyst to convert the analyte into an electrical signal [306]. They detect analytes by reducing or oxidizing them in the presence of enzyme catalysts. By electrochemical techniques, such as cyclic voltammetry or amperometry, the electrode surface redox potential can be measured [307]. Enzymatic HWEBs is the enzyme-based biosensor using enzymes as biological components due to their specificity and high reactivity. When the target molecule is present in the sample, it binds to the enzyme, leading to a conformational change that can be detected by a change in the electrical signal [308]. In recent years, electrochemical enzymatic biosensors have found applications in various fields, including clinical diagnosis, food safety, environmental monitoring, and pharmaceuticals [309].

*Glucose*

HWEBs for monitoring glucose are designed to be integrated into wearable devices, such as patches or flexible electrodes [253]. These biosensors usually use GOx as an enzyme that oxidizes glucose to gluconic acid and H_2_O_2_. The H_2_O_2_ produced can be detected electrochemically, allowing for the quantification of glucose levels in biological samples [252]. One of the approaches to designing and fabricating electrochemical enzymatic biosensors is electrode modification using conductive polymers or nanoparticles. Conductive polymers, such as PEDOT:PSS, can be used to enhance the sensitivity of biosensors. Nanoparticles like PB are electrocatalysts for Glucose conversion. The modified biosensor can improve biocompatibility, stability, and sensitivity, leading to improved glucose detection accuracy [51].

Lin et al. [253] developed a non-invasive sweat glucose sensor that utilizes hydrogel patches to rapidly collect natural perspiration without external stimulation. The hydrogel patch absorbs sweat from the hand and generates H_2_O_2_ proportional to the glucose concentration in the patch. This H_2_O_2_ is quickly reduced through a PB layer at a low overpotential while the sweat glucose sensor tracks the response signal hourly for long-term glucose monitoring. The observed signal showed a linear range of 6.25 μM to 0.8 mM and a LOD of 4 μM toward glucose concentrations with high specificity.

*Lactate*

Lactate is a common metabolic intermediate that is widely used as a marker of cellular metabolism. The most used enzyme in lactate biosensors is LOx, which converts lactate to pyruvate and H_2_O_2_. The H_2_O_2_ produced can then be oxidized at the electrode surface and change the electrical signals [310]. Enzymatic HWEBs for lactate detection are usually made by incorporating LOx into an electrochemical hydrogel, which serves as the matrix for enzyme immobilization and provides a conducive environment for the electron transfer [311]. An enzymatic biosensor electrode pad fitted to eyeglasses has been designed to measure lactate in human sweat during exercise. The biosensor displayed linearity for lactate concentrations up to 25 mM in phosphate-buffered pH 7.0 solutions. The amperometric profiles reflected changes in sweat lactate concentrations with the intensity of physical exercise when the biosensors were applied to an analysis of sweat lactate dynamics during cycling exercise [7].

*Cholesterol*

Enzymatic HWEBs for cholesterol detection can help individuals manage their health proactively and reduce the risk of cardiovascular disease By providing real-time and non-invasive monitoring of cholesterol levels [312]. The enzymes involved in this process are typically cholesterol oxidases, which catalyze the oxidation of cholesterol to cholest-4-en-3-one, generating hydrogen peroxide in the process. The hydrogen peroxide is then detected by an electrochemical transducer [313].

In a recent study, researchers reported the development of a wireless and soft smart contact lens that is capable of recording cholesterol levels in tear fluids in real time for monitoring patients with hyperlipidemia using a smartphone. In addition to an NFC chip, the sensor was also integrated with a stretchable antenna on the contact lens, allowing it to operate wirelessly and without batteries. In the soft contact lens, the silicone elastomer is used, which is biocompatible and suitable for medical applications. The protective film is a black-painted circular PI film and PDMS thin film. On a polyimide film substrate modified with cholesterol oxidase enzyme, a Cr/Au layer was deposited by thermal evaporation as a working electrode for the biosensor. Finally, The LOD of 9.91 μM was achieved for cholesterol detection [314].

*Hydrogen peroxide (H_2_O_2_)*

Hydrogen peroxide (H_2_O_2_) is a reactive oxygen species that plays an important role in various physiological and pathological processes, making it an important target for biosensor development [315]. Enzymatic electrochemical biosensors for H_2_O_2_ detection typically incorporate enzymes, such as horseradish peroxidase (HRP) or catalase, into the hydrogel matrix. These enzymes catalyze the reaction between H_2_O_2_ and a suitable electron acceptor, such as phenol, to produce water and oxygen, generating a change in the electrochemical signal [316].

A recent development Involves the creation of a self-standing electrochemical sensor that utilizes flexible hydrogels for the detection of H_2_O_2_ in liquid environments. The sensor design aimed to optimize the composition of PEDOT:PSS, hydrophilic polyurethane (HPU) hydrogel, and HRP to achieve mechanically stable sensors with desirable sensitivity and selectivity for hydrogen peroxide detection. In this design, PEDOT:PSS acts as the transducer, HPU serves as the hydrogel matrix, and HRP functions as the specific redox enzyme for H_2_O_2_. These sensors demonstrate remarkable stability, with response times of less than 6 s, and offer a detection range spanning from 100 µM to 101.6 mM [317]. The flexibility and mechanical stability of this hydrogel-based electrochemical sensor make it well-suited for wearable applications.

*Alcohol*

Alcohol dehydrogenase (ADH) and Aldehyde dehydrogenase (ALDH) are commonly used alcohol-oxidase (aOx) enzymes for alcohol detection as they catalyze the oxidation of alcohols to aldehydes and subsequently to carboxylic acids. Alcohol in the sample diffused to an electrode surface. Then is converted to acetaldehyde by ADH. In this process, acetaldehyde is further oxidized to acetic acid by ALDH, resulting in the production of electrons and protons. As electrons and protons are transferred to the electrode surface, an electrical signal proportional to the amount of alcohol in the sample is generated [318].

Researchers have developed a wearable tattoo-based alcohol biosensing system that can be used to detect alcohol levels in induced sweat without invasive measurement. Using an aOx enzyme and a printed PB electrode transducer, ethanol could be amperometrically detected in the sweat generated by the wearable prototype through transdermal delivery of the pilocarpine drug. A skin-compliant designed biosensor is highly selective and sensitive (0.362 µA mM^−1^) to ethanol [319].

#### 4.2.2. Bioaffinity HWEBs

Bioaffinity HWEBs are promising devices for non-invasive continuous monitoring of biomolecules. In such devices, bioaffinity molecules, such as antibodies or aptamers, act as bioreceptors and are coated on top of hydrogel to selectively bind to the target biomolecules [320,321,322].

*Immunosensor HWEBs*

The immunosensors, also known as antibody-based sensors, rely on the specific interaction between an antibody and an antigen to produce a change in signal transduction. Immunosensors are extremely sensitive and can detect minor concentrations of biomolecules that allow for rapid and precise analysis. When the target analyte binds to the antibody, it triggers a conformational change in the antibody that can be detected by changes in the electrochemical properties of the sensor [18].

In order to detect cytokine levels in human serum for the identification of COVID-19 as a “symptom diagnostic biomarker” and to obtain real-time information about the individual’s health status, Shi and colleagues [320] have developed a cost-effective immunosensor based on a microfluidic paper-based system. This immunosensor serves to predict the health status of COVID-19. The foldable paper-based assay employs a magnetic immunoassay and streptavidin-horseradish peroxidase combined with tetramethyl benzidine/hydrogen peroxide (TMB/H_2_O_2_) to amplify the signal for electrochemical readout. The researchers enhanced the sensitivity of cytokine detection by modifying the working electrode with a hybrid of gold nanoparticles and polypyrrole hydrogel, which increased conductivity and improved the electron transfer rate. Operating in differential pulse voltammetry mode, this paper-based immunosensor exhibited excellent performance, with a dynamic range spanning from 5 to 1000 pg mL^−1^ and a lower detection limit of 0.654 pg mL^−1^. To evaluate its clinical application, the researchers tested the proposed immunosensor using human serum samples obtained from a hospital, and the results indicated its significant potential for early diagnosis of high-risk COVID-19 patients.

*Nucleic Acid-based HWEBs*

Nucleic acids, specifically DNA and RNA, play a crucial role in maintaining the genetic information of cells and viruses [323]. Nucleic acid-based Electrochemical biosensors have the advantage of being quick to respond and can be produced inexpensively due to their simple instrumentation. In these biosensors, DNA is attached to an electrode, and the resulting hybridization reaction causes a change in electrical properties, such as current, potential, impedance, or conductance, which can be measured in the biosensors [324]. Nucleic acid-based HWEBs make use of unique properties of nucleic acids, such as specific recognition and hybridization, to detect target biomolecules [325].

Yang et al. [326] introduced a wearable epidermal system that combines reverse iontophoresis and microneedles (MNs) to improve the sensitivity and capture efficiency of cell-free DNA from ISF. The system specifically targets Epstein–Barr virus cell-free DNA, which is a significant biomarker for nasopharyngeal carcinoma diagnosis. The wearable system can extract and sense the target DNA within 10 min, with a maximum capture efficiency of 95.4% and a detection limit of 1.1 copies per µL using a recombinase polymerase amplification electrochemical microfluidic biosensor. The study also validates the feasibility of the wearable system using immunodeficient mouse models. This new approach to minimally invasive ISF sampling provides a promising pathway for cancer screening and prognosis through the detection of cell-free DNA.

*Aptamer-based HWEBs*

An aptasensor is a type of biosensor that utilizes aptamers as the recognition element [327]. Aptamers are short synthetic single-stranded oligonucleotides (e.g., DNA or RNA molecules) that are designed to bind specifically to a target molecule with a high-affinity [328]. The aptamers in electrochemical aptasensors are typically attached to a conductive material, such as gold, which allows the interaction between the aptamer and target to be monitored electrically [329]. Aptamers are capable of binding to their target molecules with a higher affinity than antibodies because they can form more stable and specific interactions with their targets. This is due to their ability to adopt various 3D structures, which enhances their ability to bind to their targets with a high degree of specificity and affinity [330].

Karuppaiah and colleagues [321] have developed a novel method for detecting low concentrations of cortisol in human saliva samples. This method utilizes an aptamer-based electrochemical sensing platform with a hybrid hydrogel network. Detecting low cortisol concentrations in saliva, which is essential for assessing physiological stress, is challenging due to the interference from salivary proteins and mucin. In this approach, the aptamer is connected to a redox probe to generate a signal, while the hydrogel network incorporates gold nanocubes to enhance electrical conductivity. This hybrid hydrogel network effectively reduces the matrix effect, enabling the detection of physiologically relevant cortisol concentrations (0.1–50 ng mL^−1^) in human saliva samples.

## 5. Challenges and Prospects

### 5.1. General Considerations

HWEBs hold immense promise for clinical diagnosis, but several scientific and technical challenges need to be addressed for widespread adoption. Technical challenges include the production cost of HWEBs, biocompatibility and robustness of hydrogels, long-term safety, miniaturization, development of sustainable and eco-friendly materials, secure and reliable wireless data transfer modules and protocols, protection against unauthorized access or misuse, suitable platform for integrating HWEBs into the skin, integration of HWEBs with other healthcare systems, power source, limited shelf-life due to biodegradation of hydrogels, and development of cost-effective and scalable manufacturing processes. Researchers are investigating novel hydrogel nanocomposites, biorecognition elements, and modern synthetic approaches to address these challenges.

The integration of biological components with HWEBs must be performed in a way that ensures seamless performance without compromising functionality. Advanced methods are needed to create complex shapes, such as microneedles with hydrogels and to control their size for optimal performance. The combination of hydrogels with microfluidic devices has the potential to revolutionize the field of HWEBs for medical and health monitoring applications. Materials used in the production of HWEBs pose challenges to ensure they are safe and efficient, with a lack of compatibility between hydrogels and electronic components still being challenging. Conductive hydrogels made of PEDOT, PANi, and PPy polymers are being investigated as they have high flexibility, proper conductivity, and stretchability and can immobilize bioreceptors, such as enzymes.

Graphene-based hydrogels and metallic and non-metallic nanomaterials, such as Au NPs and CNTs, can significantly enhance the mechanical and electrochemical performance of HWEBs. Sustainable and eco-friendly materials, such as biodegradable and renewable materials, can reduce the environmental impact of HWEBs. Microfluidic HWEBs are suitable for sweat and tear analysis, but their main challenge is the long-term collection of fluids. Tattoo-based HWEBs are more stylish and easier to use, but wearable patches are better at detecting biomarkers. Microneedles are suitable for blood analysis, but their susceptibility to infections can be addressed by using more biocompatible hydrogels.

Scientific challenges can be divided into two major categories: electrochemical and mechanical challenges. The sensing layer must be biocompatible, able to detect the target analyte and have a suitable electrical response, while the substrate must be flexible, lightweight, and durable for continuous wear. The materials used in the electrodes of these biosensors must be carefully selected to ensure high electroactivity and low interference from other biological species. For example, materials like gold and platinum are commonly used in the electrodes due to their high conductivity and stability, but their high cost and limited availability limit their widespread implementation.

To address these challenges, researchers are significantly investigating novel hydrogel nanocomposites, biorecognition elements, and modern synthetic approaches. For example, by employing genetic engineering and bioconjugation, the biological component of HWEBs can be modified to improve their specificity toward the analyte of interest. Conjugating a biological component to a nanoparticle is mostly investigated by researchers. Additionally, the manufacturing process must be compatible with large-scale production while maintaining high quality and reproducibility.

### 5.2. Electrochemical Aspects

From an electrochemical perspective, the development of HWEBs presents several challenges that must be overcome to achieve optimal performance. These challenges encompass sensitivity, specificity, long-term stability, reliability, and accuracy. Additionally, HWEBs face limitations in terms of integration with other devices and systems. Electrochemical biosensors often rely on specialized instruments, like potentiostats, for operation and result analysis, hindering their seamless integration into larger systems such as wearable health monitors or remote monitoring platforms. To tackle this issue, the miniaturization of HWEBs through microfabrication techniques and advanced materials holds promise for creating portable devices that can be easily integrated into larger systems. The miniaturization process necessitates the utilization of novel materials and fabrication techniques, including printed electronics, to develop highly sensitive and selective biosensors that can be seamlessly integrated into wearable devices. Moreover, the advent of low-cost and user-friendly instruments has simplified electrochemical analysis, enabling biosensor operation without the need for specialized equipment. Wireless biosensors, which eliminate physical connections between the biosensor and instrument, enhance usability and facilitate remote monitoring. However, transmitting data generated by HWEBs to a central location for analysis and interpretation poses challenges in remote or resource-limited environments. Therefore, integrating HWEBs with smartphones and portable devices can enable convenient data acquisition and analysis, thereby facilitating integration into larger systems like wearable health monitors. Ultimately, fostering collaborative efforts between researchers and industry can bridge the gap between research and commercialization, resulting in the development of practical biosensors that seamlessly integrate into larger systems.

An inherent challenge for HWEBs lies in their susceptibility to interference from skin-related factors, such as sweat, oil, and pH changes, as they are often attached to the skin. Researchers are actively exploring new materials and techniques to enhance the performance of HWEBs in addressing these challenges. Sensitivity can be improved by employing advanced electrodes with high surface area, such as CNTs, graphene, MXenes, and MOFs, which facilitate enhanced electron transfer processes. Conversely, sensitivity may be limited by factors including low target molecule concentrations, interference, and the characteristics of the bioreceptor, such as type, size, and orientation.

Specificity can be enhanced by utilizing specific recognition elements, such as enzymes, antibodies, or aptamers, which selectively bind to the target analyte to generate a signal. However, this approach can lead to non-specific interactions with other molecules in the sample, resulting in false positive or negative results and reduced accuracy. Additionally, hydrogels, while versatile, can interact with a wide range of substances, causing interference and cross-reactivity with other components in the sample, such as proteins. This compromises the selectivity of HWEBs and complicates analysis in real biological samples.

Various factors impact the stability of the signal in HWEBs, including the choice of biorecognition elements, electrode design, and operational conditions. Harsh operational conditions, such as exposure to biofouling species, high temperatures, humidity, and mechanical stress, can lead to the degradation of biosensors over time. Moreover, materials employed in HWEBs must withstand the body’s harsh environment, maintain their electrical and mechanical properties after multiple uses and washing, and ensure secure attachment to the skin or clothing to prevent damage or loss. Collaborative efforts between researchers and industry can bridge the gap between research and commercialization, leading to the development of more practical biosensors that integrate into larger systems.

The stability of biorecognition elements is a major challenge affecting the sensitivity and specificity of HWEBs. Enzymes and antibodies are particularly unstable, and researchers have explored various strategies to enhance their stability, such as cross-linked enzyme aggregates, encapsulation in protective materials, and microfluidics. However, hydrogels used for stabilization may cause corrosion and misalignment of electrodes, reducing the accuracy and reliability of HWEBs. Hydrogels are also susceptible to physical and chemical degradation, potentially releasing harmful substances.

The evaporation of water from the hydrogel can cause several issues, including changes in conductivity, electrochemical performance, and mechanical properties. The presence of water is vital for the ionic conductivity of hydrogels, especially when they are utilized as electrolytes in electrochemical biosensors. As water evaporates, the ionic mobility within the hydrogel can be affected, leading to changes in the overall conductivity. The evaporation of water can influence the transport of ions and molecules within the hydrogel, impacting the electrochemical reactions at the electrode-hydrogel interface. This could affect the sensitivity and reliability of the biosensor. To address these challenges and enhance the long-term stability of hydrogels in air environments, state-of-the-art research has focused on various strategies, including crosslinking and polymer Structure (Section 2.1), hydration layer coatings, composite hydrogels (Section 2.2), encapsulation techniques (Section 2.3.2), smart hydrogels, and humidity control. Some studies have investigated the use of hydration layer coatings to protect the hydrogel from direct exposure to the air. These coatings create a barrier that slows down the evaporation process and preserves the hydrogel’s water content. Researchers are also exploring the development of “smart” hydrogels that can respond to environmental changes, such as water loss, by contracting or expanding to maintain their structure and properties. In some applications, it may be possible to control the surrounding humidity to minimize water evaporation from the hydrogel.

Response and recovery time are important electrochemical properties to consider for HWEBs and can be optimized through sensor design and operational conditions. Rigorous testing and validation are necessary before widespread use, and the integration of machine learning algorithms is expected to improve accuracy and reliability. The selection of the transduction principle is also critical, with amperometry being commonly used. Pulse techniques can enhance sensitivity by eliminating non-faradic current, while EIS has the lowest LOD compared to other techniques.

### 5.3. Mechanical Aspects

The mechanical properties of HWEBs are important for both user comfort and device performance. HWEBs need to be flexible to conform to the wearer’s skin and to withstand movement for long-term use. However, hydrogels have low mechanical strength, limited flexibility and tend to induce skin irritation. To solve these issues, several strategies have been explored, such as incorporating a hydrogel made from a mixture of chitosan and gelatin, which has excellent mechanical strength and biocompatibility. Moreover, HWEBs must function for extended periods of time without needing to be recharged or replaced and withstand regular wear and tear.

Environmental factors, such as temperature and humidity, can affect the mechanical behavior of hydrogels and result in unreliable and inaccurate readings. Water acts as a plasticizer, contributing to the flexibility and mechanical properties of hydrogels. As water evaporates, the hydrogel may become stiffer and more brittle, potentially compromising its structural integrity and functionality. By addressing water evaporation and its effects on electrical, electrochemical, and mechanical properties, researchers are striving to unlock the full potential of hydrogels in cutting-edge technologies. Achieving a balance between mechanical properties, swelling behavior, and ionic conductivity while maintaining biocompatibility and long-term stability is a challenging task. The use of 2D materials, such as MXene and graphene, to fabricate thin hydrogels can improve flexibility. However, controlling their thickness, mechanical performance, and electrochemical properties remains a challenge. Hydrogels are susceptible to cracking, tearing, and stretching, especially under high strains and repeated cycles of deformation, which can affect the overall performance of HWEBs. Achieving consistent mechanical performance from batch to batch is also challenging, as the mechanical behavior of hydrogels can be influenced by various factors during preparation.

To tailor the mechanical properties of hydrogels, several approaches can be employed, such as using crosslinking type and density, nano- or micro-fillers, DN hydrogels, metal ions, slide-ring hydrogel, macromolecular microsphere composite hydrogel, and supramolecular hydrogels. However, some of these methods or materials may affect other features of hydrogels, such as biocompatibility or fatigue behavior. For example, utilizing chemical crosslinkers can influence the biocompatibility of the hydrogel. Therefore, hydrogels with no chemical crosslinker can be useful in designing HWEBs. Moreover, hydrogel nanocomposites that have high strain strength may suffer from viscoelastic behavior and fatigue under working conditions, limiting reliable and accurate detection. To address this, anti-fatigue hydrogels can be useful due to the abundance of reversible and physical bonds in their networks, such as polyampholyte materials that endow hydrogels with recoverability and self-healing ability.

## 6. Conclusions

Hydrogel-based wearable electrochemical biosensors (HWEBs) have gained immense popularity for their remarkable versatility in monitoring a wide range of biometrics, including glucose, lactate, pH, and other biomarkers. In this review, we delved into the realm of hydrogels and their composites that are ideally suited for wearable electrochemical biosensors. We thoroughly examined their physical and biological properties, focusing particularly on their electrochemical and mechanical properties, and shed light on the latest advances in this field. In addition, we explored the various platforms and applications of HWEBs, which offer a non-invasive means of monitoring an individual’s metabolic activity and providing real-time data. We also highlighted the potential clinical applications of HWEBs, including the management of diabetes, the diagnosis of cancer, and the monitoring of wound healing. This technological innovation enables healthcare professionals to make informed decisions and develop personalized treatment plans. With the power to revolutionize healthcare, HWEBs represent a promising avenue for the future of biosensor technology.

## Figures and Tables

**Table 2:** Electrochemical properties of wearable electrochemical biosensors.

Materials			Biosensor Properties							Ref.
Sensing Element @ Hydrogels	Bioreceptor	Substrate	Target	Real Sample	Electrolyte	Measurement Technique	LOD (Unit)	Linear Range (Unit)	Sensitivity (Unit)	
CNs ^1^@ PAM	AOx	Glass	Ethanol	Breath	PBS	Amperometry	1 (μM)	10 to 100 (μM)	12 (mV/decade log(C))	[251]
PB/Carbon @ HEMA	GOx	PTFE coated glass	Glucose	Blood	PBS		7.9 (μM)	0 to 210 (μM)	9.4 (μA/cm^2^·mM)	[252]
Fe^3+^/PB @ PEDOT	GOx	SPCE	Glucose	Sweat	PBS		4 (μM)	6.25 to 800 (μM)	_	[253]
PEDOT:PSS	UOx ^2^	PET foil	Uric acid	wound	PBS		4.5 (μM)	50 to 1000 (μM)	_	[248]
DF ^3^/PB@ PEDOT:PSS	GOx	GCE	Glucose	Rabbit serum	PBS		0.85 (μM)	1 to 243 (μM)	340.1 (μA/cm^2^·mM)	[51]
PAM@CS	_	_	O_2_	Breath	_		5.7 (ppm)	0–100 (%)	0.2 (%/ppm)	[254]
Pt/Gr@CS	GOx	PU sheet	Glucose	Sweat	PBS		10 (μM)	0 to 900 (μM)	105 (μA/cm^2^·mM)	[255]
PtNP/AuNP/rGO/CS	GOx	Polyimide	Glucose	Sweat	PBS		5 (μM)	0 to 2400 (μM)	48 (μA/cm^2^·mM)	[256]
PB/(GO@CS)	Lox ^4^	SPCE	Lactate	Sweat	Artificial sweat		28 (nM)	0.068 to 50,000 (μM)	0.39 (μA/cm^2^·mM)	[257]
PB/(GO-CS)	GOx		Glucose				6.7 (nM)	0.032 to 3800 (μM)	8.20 (μA/cm^2^·mM)	
PVA/β-CD ^5^	GOx	SPCE	Glucose	_	PBS	Cyclic voltammetry	51 (nM)	1 to 5 (mM)	7.58 (μA/mM)	[258]
PVA/BTCA ^6^/β-CD/AuNPs	GOx	SPCE	Glucose	_	PBS		10 (μM)	0.1 to 0.5 (mM)	47.2 (μA/mM)	[71]
PB/Au@ Graphene	GOx	_	Glucose	Sweat	_		10 (μM)	10 to 700 (μM)	_	[259]
AuNPs@Peptide	_	GCE	*S. aureus*, *E. coli* and *P. aeruginosa*	_	PBS	DPV	21 (nM)	0.1 to 10 (μM)	_	[250]
MXene@BSA ^7^	Peptide	GCE	Immunoglobulin G	Serum	PBS		23 (pg/mL)	0.0001 to 10 (µg/mL)	_	[260]
Agarose/Carbon/PB ink	_	PET	MPOx	_	PBS	SWV	200 (μM)		_	[249]
Graphene/Pyrene	GOx	Si wafer	Glucose	Tears	PBS	Conductometry	12.57 (μM)	_	22.72 (%/mM)	[261]
Graphene	Cortisol Mab	SiO_2_ wafer	Cortisol	Tears	PBS		10 (pg/mL)	1 to 40 (ng/mL)	1.84 (ng/mL. %)	[262]
Au 3D nanostructure	Cortisol Ab	PDMS	Cortisol	Sweat	PBS	EIS	1 (pg/mL)	_	0.25 (Ohm/ng mL)	[263]
Ti_3_C_2_T_x_ Mxene/LBG ^8^	Cortisol Ab	Polyamide	Cortisol	Sweat	_		88 (pM)	0.001 to 100 (mM)	_	[264]
PAM/Ca-Alg ^9^	_	Thermoplastic polyurethane	pH	Sweat	_	Potentiometry	3.05		58.14 mV/pH	[265]
			Na^+^				7.94 (μM)	5 to 160 (mM)	58.89 mV/decade	
			K^+^				5.37 (μM)	1 to 32 (mM)	59.11 mV/decade	
Chitosan/Nafion	GOx	Pt	Glucose	_	DPBS		_	1–10, 10–100, 100–3000 (μM)	0.5, 0.012, 0.19 (mV/μM)	[266]

^1^ Chlorella-derived layered carbon nanosheets; ^2^ Urate oxidase; ^3^ Dimethyl sulfoxide (DMSO) and Zonyl FS-300; ^4^ Lactate oxidase; ^5^ β-cyclodextrin; ^6^ 1,2,3,4-butanetetracarboxylic acid; ^7^ Bovine serum albumin; ^8^ Laser-burned graphene; ^9^ polyacrylamide/calcium alginate hydrogel.
